# Type I interferon and mitochondrial dysfunction are associated with dysregulated cytotoxic CD8^+^ T cell responses in juvenile systemic lupus erythematosus

**DOI:** 10.1093/cei/uxae127

**Published:** 2024-12-25

**Authors:** Anna Radziszewska, Hannah Peckham, Restuadi Restuadi, Melissa Kartawinata, Dale Moulding, Nina M de Gruijter, George A Robinson, Maryam Butt, Claire T Deakin, Meredyth G Ll Wilkinson, Lucy R Wedderburn, Elizabeth C Jury, Elizabeth C Rosser, Coziana Ciurtin

**Affiliations:** Centre for Adolescent Rheumatology Versus Arthritis at UCL, UCLH, GOSH, London, UK; Department of Ageing, Rheumatology & Regenerative Medicine, Division of Medicine, UCL, London, UK; Centre for Adolescent Rheumatology Versus Arthritis at UCL, UCLH, GOSH, London, UK; Department of Ageing, Rheumatology & Regenerative Medicine, Division of Medicine, UCL, London, UK; Centre for Adolescent Rheumatology Versus Arthritis at UCL, UCLH, GOSH, London, UK; Infection, Immunity and Inflammation Research & Teaching Department, UCL Great Ormond Street Institute of Child Health, London, UK; Centre for Adolescent Rheumatology Versus Arthritis at UCL, UCLH, GOSH, London, UK; Infection, Immunity and Inflammation Research & Teaching Department, UCL Great Ormond Street Institute of Child Health, London, UK; NIHR Biomedical Research Centre at Great Ormond Street Hospital, London, UK; Developmental Biology and Cancer Research & Teaching Department, UCL Great Ormond Street Institute of Child Health, London, UK; Centre for Adolescent Rheumatology Versus Arthritis at UCL, UCLH, GOSH, London, UK; Department of Ageing, Rheumatology & Regenerative Medicine, Division of Medicine, UCL, London, UK; Centre for Adolescent Rheumatology Versus Arthritis at UCL, UCLH, GOSH, London, UK; Department of Ageing, Rheumatology & Regenerative Medicine, Division of Medicine, UCL, London, UK; Centre for Adolescent Rheumatology Versus Arthritis at UCL, UCLH, GOSH, London, UK; Department of Ageing, Rheumatology & Regenerative Medicine, Division of Medicine, UCL, London, UK; Centre for Adolescent Rheumatology Versus Arthritis at UCL, UCLH, GOSH, London, UK; Infection, Immunity and Inflammation Research & Teaching Department, UCL Great Ormond Street Institute of Child Health, London, UK; NIHR Biomedical Research Centre at Great Ormond Street Hospital, London, UK; Centre for Adolescent Rheumatology Versus Arthritis at UCL, UCLH, GOSH, London, UK; Infection, Immunity and Inflammation Research & Teaching Department, UCL Great Ormond Street Institute of Child Health, London, UK; NIHR Biomedical Research Centre at Great Ormond Street Hospital, London, UK; Centre for Adolescent Rheumatology Versus Arthritis at UCL, UCLH, GOSH, London, UK; Infection, Immunity and Inflammation Research & Teaching Department, UCL Great Ormond Street Institute of Child Health, London, UK; NIHR Biomedical Research Centre at Great Ormond Street Hospital, London, UK; Department of Ageing, Rheumatology & Regenerative Medicine, Division of Medicine, UCL, London, UK; Centre for Adolescent Rheumatology Versus Arthritis at UCL, UCLH, GOSH, London, UK; Department of Ageing, Rheumatology & Regenerative Medicine, Division of Medicine, UCL, London, UK; Centre for Adolescent Rheumatology Versus Arthritis at UCL, UCLH, GOSH, London, UK; Department of Ageing, Rheumatology & Regenerative Medicine, Division of Medicine, UCL, London, UK

**Keywords:** CD8^+^ T cells, cytotoxicity, juvenile systemic lupus erythematosus, interferon

## Abstract

Juvenile systemic lupus erythematosus (JSLE) is an autoimmune condition which causes significant morbidity in children and young adults and is more severe in its presentation than adult-onset SLE. While many aspects of immune dysfunction have been studied extensively in adult-onset SLE, there is limited and contradictory evidence of how cytotoxic CD8^+^ T cells contribute to disease pathogenesis and studies exploring cytotoxicity in JSLE are virtually non-existent. Here, we report that CD8^+^ T cell cytotoxic capacity is reduced in JSLE versus healthy controls, irrespective of treatment or disease activity. Transcriptomic and serum metabolomic analysis identified that this reduction in cytotoxic CD8^+^ T cells in JSLE was associated with upregulated type I interferon (IFN) signalling, mitochondrial dysfunction, and metabolic disturbances when compared to controls. Greater interrogation of the influence of these pathways on altered cytotoxic CD8^+^ T cell function demonstrated that JSLE CD8^+^ T cells had enlarged mitochondria and enhanced sensitivity to IFN-α leading to selective apoptosis of effector memory (EM) CD8^+^ T cells, which are enriched for cytotoxic mediator-expressing cells. This process ultimately contributes to the observed reduction in CD8^+^ T cell cytotoxicity in JSLE, reinforcing the growing evidence that mitochondrial dysfunction is a key pathogenic factor affecting multiple immune cell populations in type I IFN-driven rheumatic diseases.

## Teaser

Juvenile systemic lupus erythematosus (JSLE) is a severe autoimmune disease affecting children and young people. Here we report that frequencies of perforin, IFN-γ and TNF-α-expressing CD8+ T cells are reduced in JSLE compared with healthy controls. The reduction in CD8+ T cell cytotoxic potential in JSLE is accompanied by enlargement of CD8+ T cell mitochondria and increased susceptibility to apoptosis, which leads to a deficiency in effector memory (EM) CD8+ T cells, all of which are associated with increased type I interferon.

## Graphical Abstract

**Graphical Abstract:**
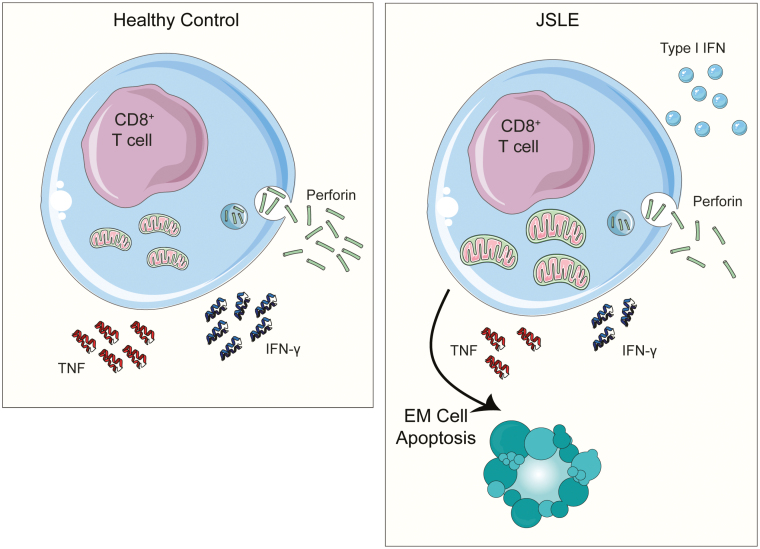

## Introduction

Systemic lupus erythematosus (SLE) is a complex, multisystem autoimmune condition characterized by production of autoantibodies to nuclear antigens. Disease presentation, clinical course, and outcome may vary considerably between individuals, age groups, and ethnicities. There are numerous clinical manifestations which range from mild articular and cutaneous involvement to life-threatening manifestations, such as central nervous system, cardio-pulmonary or renal involvement, or catastrophic thrombosis, particularly in individuals with associated anti-phospholipid syndrome [1].

If SLE develops before the age of 18, it is classified as juvenile systemic lupus erythematosus (JSLE), which accounts for approximately 15–20% of all SLE cases [2]. JSLE is characterized by more aggressive disease, widespread organ involvement, and worse outcomes compared to adult-onset disease [3, 4]. Importantly, differences between JSLE and adult-onset SLE continue into adulthood in patients with juvenile-onset disease [5, 6]. Despite this, commonly used treatments, primarily relying on broad immunosuppression with corticosteroids and conventional disease-modifying antirheumatic drugs (DMARDS), remain the same and do not account for age-specific disease differences.

Cytotoxic T lymphocytes (CD8^+^ T cells) are an important component of the adaptive immune system involved in infection control and cancer immunosurveillance [7]. They recognize foreign peptides that are presented on major histocompatibility complex class I (MHC-I) molecules on antigen presenting cells. CD8^+^ T cells exert their effector function in three ways: by releasing granules filled with cytotoxic molecules, through Fas (CD95)/FasL (Fas ligand) interactions, and by releasing effector cytokines [8]. The main mechanism by which cytotoxic CD8^+^ T cells kill pathogen-infected or tumorigenic cells is through calcium-dependent release of lytic granules loaded with cytotoxic enzymes such as perforin and granzymes. CD8^+^ T cells can also induce target cell death via direct Fas/FasL and TNF-related apoptosis-inducing ligand (TRAIL)/TRAIL-R interactions [9]. Both the fast-acting perforin-based mechanism and the slower Fas-based mechanism of cytotoxic cell death can occur at the same time, but their regulation is thought to be distinct [10, 11]. In addition, activated CD8^+^ T cells can mediate target cell death by producing antiviral and pro-inflammatory cytokines such as IFN-γ, TNF-α, and IL-2.

In animal models of lupus, mice lacking MHC-I molecules are protected from disease development and fail to generate antibodies to double-stranded DNA or nuclear antigens, suggesting a possible role for CD8^+^ T cells in SLE pathogenesis [12]. In contrast, perforin-deficient lupus-prone mice exhibit accelerated disease progression [13] and in graft-vs.-host murine lupus models both Fas and perforin are required for effective clearance of autoreactive B cells [14, 15], implicating CD8^+^ T cell cytolytic functions in maintenance of peripheral tolerance and halting autoimmunity. While increased CD8^+^ T cell effector function has been reported in adult-onset SLE, particularly in active disease [16–20], a large body of research also suggests a defect in effector function [21–28]. Other CD8^+^ T cell functional abnormalities described in adult-onset SLE include impaired Ebstein-Barr Virus (EBV)-specific responses [29–31], metabolic abnormalities linked to mitochondrial dysfunction [32, 33], and an upregulation of type I IFN-stimulated genes [20, 34].

Recent research from our laboratory, in which a machine learning approach was used to analyse immune phenotypes in a large cohort of JSLE patients, has emphasized the need for a more in-depth characterization of CD8^+^ T cell phenotype and function in JSLE [35]. Interestingly while no differences in B cell subsets were observed within the patient cohort, differences in CD8^+^ T cell subsets could be used to stratify patients with JSLE. The fact that JSLE patients can be stratified by CD8^+^ T cell phenotype highlights the heterogeneity of this patient group and this may, at least in part, account for the disparate findings reported by various studies worldwide.

Despite growing evidence indicating that CD8^+^ T cells may play a role in the pathology and disease progression in adult-onset SLE, very little research has focused on their role in JSLE [7]. In the present study, to address this, using flow cytometric and transcriptomic analysis we have undertaken an in-depth exploration of CD8^+^ T cell cytotoxic capacity in peripheral blood in a large cohort of clinically well-characterized JSLE patients alongside age- and sex-matched healthy controls. We demonstrate that CD8^+^ T cell cytotoxic capacity is reduced in JSLE irrespective of disease activity or treatment and may be linked to IFN-induced apoptosis and abnormalities in mitochondrial function and non-lipid serum metabolome. This study represents an in-depth investigation of CD8^+^ T cell cytotoxicity in a unique cohort of JSLE, which could have important implications for elucidating the potential role of CD8^+^ T cells in JSLE pathogenesis.

## Materials and methods

### Patient and control samples

All research participants were recruited with informed age-appropriate consent as approved by the London-Harrow Research Ethics Committee (study reference: 11/LO/0330) and research was conducted in accordance with the Declaration of Helsinki and NHS HRA (National Health Service Health Research Authority) guidelines as summarized in the ethical approval statement. Patients diagnosed with JSLE were recruited from the adolescent rheumatology clinics at University College London Hospital (UCLH). Healthy controls (HC) aged 15–16 years old with no prior history of autoimmune disease were recruited from pre-assessment dental and urological surgery clinics at UCLH. Blood from these participants was taken under general anaesthetic at the time of surgery. HC samples from young people aged 16 and over were obtained from volunteers from the community. Participants were excluded if they had viral symptoms or received any vaccine in the previous 3 weeks. 44 JSLE patients and 68 age-matched HC were included in the study. Clinical and demographic data for all participants is shown in Table 1. Detailed clinical characteristics and pathology marker data were obtained from patient medical records.

**Table 1:** Demographic and clinical characteristics of healthy controls and JSLE patients in phenotyping cohort

	Healthy controls Number (%/range)	JSLE Number (%/range)	*P*-value
Total number	68	44	-
Female:male	46:22	34:10	0.29
Median age (years)	20.1 (15.2–32.2)	21.6 (15.6–29.8)	0.27
Ethnicity (%)			
White	34 (50%)	18 (41%)	0.44
South Asian	12 (18%)	13 (30%)	0.17
East Asian	11 (16%)	3 (7%)	0.24
Black	5 (7%)	6 (13%)	0.34
Other	6 (9%)	4 (9%)	1.00
Clinical features^a^^,^^b^		Number (%/range)	
Median disease duration (years)		8.9	-
Median age at onset (years)		12.3	-
Average SLEDAI, *n* = 40		1.6 (0–10)	-
SLEDAI = 0–4		40 (91%)	-
SLEDAI = 6–10		4 (9%)	-
Average BILAG		1.0 (0–9)	-
Global BILAG = 0		35 (80%)	-
Global BILAG =1 (1 score C)		4 (9%)	-
Global BILAG = 8 (1 score B)		3 (7%)	-
Global BILAG = 9 (1 score B + 1 score C)		2 (5%)	-
Renal involvement		15 (34%)	-
Constitutional involvement		15 (34%)	-
Neuropsychiatric involvement		8 (18%)	-
Mucocutaneous involvement		38 (86%)	-
Musculoskeletal involvement		29 (66%)	-
Haematological involvement		33 (75%)	-
Cardiorespiratory involvement		7 (16%)	-
Gastrointestinal involvement		1 (2%)	-
Ophthalmic involvement		0 (0%)	-
Serology^a^		Median	
Anti-dsDNA (IU/mL) (NR=<50), *n* = 39		26.0	-
C3 (g/L) (NR=0.9–1.8), *n* = 40		1.10	-
Lymphocyte count (10^9^/L) (NR=1.2–3.5)		1.68	-
Leukocyte count (10^9^/L) (NR=3–10), *n* = 37		5.79	-
Treatment^a^		Number (%)	
None		3 (7%)	-
Rituximab in the past year		0 (0%)	-
Rituximab ever		12 (27%)	-
Average duration since last rituximab treatment (years)		4.7	-
Hydroxychloroquine		38 (86%)	-
Methotrexate		5 (11%)	-
Azathioprine		8 (18%)	-
Mycophenolate mofetil		19 (43%)	-
Prednisolone (any dose)		10 (23%)	-
Prednisolone ≥ 10mg/day		6 (14%)	-
Cyclophosphamide in the past year		1 (2%)	-

*P*-values calculated using Mann–Whitney U test (age) or Fisher’s exact test (sex and ethnicity). anti-dsDNA: anti-double-stranded DNA antibody; BILAG: British Isles Lupus Activity Group global score; C3: complement 3; HC: healthy controls; JSLE: juvenile systemic lupus erythematosus; NR: normal range; SLEDAI: Systemic Lupus Erythematosus Disease Activity Index-2K.

^a^Data is presented for the entire JSLE cohort (*n* = 44), unless stated otherwise.

^b^Organ involvement includes patients with current or previous disease activity in the specified organ domain.

Disease scores were calculated using SLE Disease Activity Index 2000 (SLEDAI-2K) and British Isles Lupus Assessment Group (BILAG) scores by physicians at the time of clinical visit. Active disease was defined as a SLEDAI score > 4 or global BILAG score ≥8.

### PBMC and serum isolation from blood

#### PBMC isolation

PBMCs were isolated by Ficoll gradient centrifugation using SepMate^TM^ (StemCell) tubes, as described [36]. Viable cells were counted by trypan blue exclusion and cryopreserved in 10% DMSO, 90% FBS freezing media for long-term storage in vapour phase liquid nitrogen.

#### Serum isolation

Whole blood collected in SST II Vacutainer tubes (BD Biosciences) was centrifuged at 12 000*g* for 10 min to separate the serum from the remainder of the blood. The separated serum was then aliquoted and stored at −80°C.

### Flow cytometry

PBMC populations were phenotyped by multi-parameter flow cytometry using commercially available fluorochrome conjugated antibodies. PBMCs were thawed in complete media consisting of RPMI-1640, 10% FBS, penicillin (100 IU/ml) and streptomycin (100 μg/ml) and plated in 96-well plates at a density of 0.5 × 10^6^/well. The average post-thaw cell viability was 74.4%. All subsequent steps were performed at room temperature (RT) with incubations performed in the dark. During wash steps, the plates were centrifuged at 500g for 5 min.

#### Surface staining

To exclude dead cells, PBMCs were stained with UV Live/Dead fixable blue stain (Thermo Fisher Scientific) or Ghost Dye Violet 510 (Tonbo Biosciences) for 15 min. This was followed by a wash in FACS buffer (phosphate buffered saline (PBS) + 1% FBS + 2mM EDTA). Surface staining was performed by incubating with the following antibodies in FACS buffer or Brilliant™ Stain Buffer (BD Biosciences) for 20 min: Panel 1—T cells: BV605-CD3 (BioLegend Cat# 317322, RRID:AB_2561911), AF700-CD8 (BioLegend Cat# 344723, RRID:AB_2562789), APC-Cy7-CD4 (BioLegend Cat# 317418, RRID:AB_571947), PERCP-Cy5.5-CD45RO (BioLegend Cat# 304222, RRID:AB_2174124), PE-CCR7 (BioLegend Cat# 353203, RRID:AB_10916391); Panel 2—Senescence: BUV805-CD3 (BD Biosciences Cat# 612895, RRID:AB_2870183), APC-Cy7-CD4 (BioLegend Cat# 317418, RRID:AB_571947), AF700-CD8 (BioLegend Cat# 344723, RRID:AB_2562789), BV421-CD45RA (BioLegend Cat# 304129, RRID:AB_10900421), PE-Cy7-CCR7 (BioLegend Cat# 353225, RRID:AB_11125576), BV711-CD27 (BioLegend Cat# 302833, RRID:AB_11219201), BV785-CD28 (BioLegend Cat# 302949, RRID:AB_2629585), PE-KLRG1 (Miltenyi Biotec Cat# 130-120-566, RRID:AB_2784406); Panel 3—Cytotoxicity: BUV805-CD3 (BD Biosciences Cat# 612895, RRID:AB_2870183), BV711-CD4 (BioLegend Cat# 317440, RRID:AB_2562912), BV421-CD8 (BioLegend Cat# 344747, RRID:AB_2629583), PE-CCR7 (BioLegend Cat# 353203, RRID:AB_10916391), PE-Dazzle-CD45RO (BioLegend Cat# 304247, RRID:AB_2566542); Panel 4—Cytokines: BUV805-CD3 (BD Biosciences Cat# 612895, RRID:AB_2870183), APC-Cy7-CD4 (BioLegend Cat# 317418, RRID:AB_571947), AF700-CD8 (BioLegend Cat# 344723, RRID:AB_2562789), PerCP-Cy5.5-CD45RO (BioLegend Cat# 304222, RRID:AB_2174124), BV421-CCR7 (BioLegend Cat# 353207, RRID:AB_10915137), BUV737-PD-1 (BD Biosciences Cat# 565299, RRID:AB_2739167). After a wash step, if intracellular staining was not required, cells were fixed by incubation for 15 min in 2% paraformaldehyde and washed twice in FACS buffer.

#### Intracellular staining

For intracellular staining, cells were incubated for 20 min in fixation buffer (ebioscience^TM^Foxp3/Transcription Factor Staining Buffer Set, Thermo Fisher, 00-5523-00). Cells were then washed in permeabilization buffer and incubated for 40 min with the following intracellular antibodies in permeabilization buffer: Panel 3—Cytotoxicity: FITC-Granzyme A (BioLegend Cat# 507204, RRID:AB_315470), PerCP-Cy5.5-Perforin (BioLegend Cat# 353314, RRID:AB_2571971), AF700-Granzyme B (BioLegend Cat# 372221, RRID:AB_2728388), and PE-Granulysin (instead of PE-CCR7 in some experiments); Panel 4—Cytokines: BV510-TNF-α (BioLegend Cat# 502949, RRID:AB_2565859) and BV605-IFN-γ (BioLegend Cat# 502535, RRID:AB_11125368). This was followed by a wash in permeabilization buffer and a final wash in FACS buffer.

#### Data acquisition and analysis

Data were acquired using a BD LSR II Flow Cytometer (RRID:SCR_002159) running BD FACSDiva Software (RRID:SCR_001456). As many events as possible were acquired. Data were analysed using FlowJo software (TreeStar, RRID:SCR_008520). Population frequencies were expressed as percentage of parent population in all analyses, unless indicated otherwise.

### Primary cell culture assays

#### Cytokine detection

To stimulate cytokine production, PBMCs (0.5 × 10^6^ cells/well) were incubated with phorbol 12-myristate 13-acetate (PMA, 50 ng/mL, Sigma), ionomycin (250 ng/mL, Sigma) brefeldin A (5 µg/mL, BioLegend) and cultured in complete media at 37°C in 5% CO_2_ for 4 h. Subsequent to incubation with stimulants, cells were stained as described above, with fluorochrome conjugated antibodies listed in Panel 4.

#### Degranulation assay

To assess the degranulation capacity of CD8^+^ T cells, in a subset of HC and JSLE patients, PBMCs (0.5 × 10^6^ cells/well) were incubated in complete media with PMA, ionomycin, brefeldin A as described above and by Betts et al. [37, 38]. In addition, monensin (5 ug/mL) was added at the same time to neutralize the acidic pH of the endosome and prevent degradation of endocytosed fluorochrome conjugated CD107a antibody [39]. For staining of the degranulation marker CD107a, PE-CD107a antibody (BioLegend Cat# 328608, RRID:AB_1186040) was added at the same time as the stimulants. After 4-h incubation, cells were stained with remaining antibodies in Panel 4.

#### Antigenic peptide stimulation assay

PBMCs (1 × 10^6^ per well) were incubated in complete media with a peptide pool of MHC-I restricted antigens (PepMix CEFX Ultra SuperStim MHC-I Subset, 5µg/mL, JPT, PM-CEFX-4) or dimethyl sulfoxide (DMSO) (as an unstimulated control) for 6 h at 37°C in 5% CO_2_ in the presence of brefeldin A (5 µg/mL), monensin (5 µg/mL), and PE-CD107a antibody (BioLegend Cat# 328608, RRID:AB_1186040). After incubation, cells were stained as described above with the following surface antibodies: BUV395-CD3 (BD Biosciences Cat# 563546, RRID:AB_2744387) and BV785-CD8 (BioLegend Cat# 344739, RRID:AB_2566201), and intracellular BV421-IFN-γ (BioLegend Cat# 502531, RRID:AB_10900083).

#### Apoptosis assay

Thawed PBMCs (0.5 × 10^6^ cells/well) from JSLE patients and HC were incubated in complete media or in complete media containing 1000 IU/ml of IFN-α2b [40] for 48 h [32] prior to surface staining with antibodies BUV395-CD3 (BD Biosciences Cat# 563546, RRID:AB_2744387), BV711-CD4 (BioLegend Cat# 317439, RRID:AB_11219404), BV785-CD8 (BioLegend Cat# 344739, RRID:AB_2566201), BV421-CD45RO (BioLegend Cat# 304223, RRID:AB_10898323), and APC-Cy7-CCR7 (BioLegend Cat# 353213, RRID:AB_10915474) for 20 min. The IFN-α2b subtype was chosen as it is a known potent inducer of several type I IFN-stimulated genes [41]. To detect apoptosis using the FITC Annexin V Apoptosis Detection Kit (BD Biosciences, Cat# 556547), cells were washed in binding buffer and stained with Annexin V and propidium iodide (PI) for 15 min as per manufacturer’s protocol. Acquisition of flow cytometric data on unfixed cells was performed within 1 h of staining.

### Imaging

Imaging was performed as previously described by Wilkinson et al. [42] In summary, live cells were imaged using a Zeiss Axio Observer 7 microscope (RRID:SCR_023694) with a 63xNA1.4 Oil objective and Hamamatsu Flash 4 camera. PBMCs freshly isolated from whole blood were stained with APC-CD8 (BioLegend Cat# 344722, RRID:AB_2075388) in FACS buffer for 20 min followed by incubation with MitoTracker Green (Thermo Fisher Scientific, M7514) for 30 min at 37°C in 1× PBS and left unfixed. Cells were suspended in FACS buffer in 96-well glass bottom imaging plates with a #1.5 coverslip base. LED illumination at 470nm or 630nm with single band filters (APC-CD8 and MitoTracker Green (excitation/emission 490/516 nm)) were used to take z-stacks at Nyquist sampling in *x*, *y*, and *z*. Exposure times (20 ms) and illumination intensity were kept to minimal levels to avoid phototoxicity and allow rapid imaging of each cell. Typically, 500 grey levels (on a 16 bit detector) were used for each channel. Individual cells were manually cropped using Fiji software [43], deconvolved with Huygens Software (RRID:SCR_014237), then processed with in-house macros (MitoSoxWithTablev016.ijm at Fiji-Macros/README.md at master · DaleMoulding/Fiji-Macros (github.com)) to measure MitoTracker volume and surface area within a cropped spheroid around each cell. Stacked images were flattened for visualization in Fiji by Z-projection using the standard deviation projection method. 3D model images were created using Imaris cell imaging software (RRID:SCR_007370) (Oxford Instruments).

### Cell sorting and RNA isolation

Thawed PBMCs from 29 HC and 26 JSLE patients were stained with Zombie NIR™ Fixable dye (1µl/30 million cells, BioLegend, 423105) followed by BUV395-CD4 (BD Biosciences Cat# 563550, RRID:AB_2738273), APC-CD3 (BioLegend Cat# 300412, RRID:AB_314066), BV785-CD8 (BioLegend Cat# 301045, RRID:AB_11219195), AF488-CD19 (BioLegend Cat# 302219, RRID:AB_389313), and PE-Cy7-CD14 (BioLegend Cat# 301814, RRID:AB_389353) antibodies as previously described. CD8^+^ T cells were isolated by fluorescence-activated cell sorting (FACS) using a BD Biosciences FACSAria III Cell Sorter (RRID:SCR_016695). RNA was extracted from CD8^+^ T cells using a PicoPure RNA isolation kit (Thermo Fisher Scientific, KIT0204) according to manufacturer’s instructions.

### RNA sequencing (RNA-seq)

RNA quality control, library preparation, and sequencing was performed by UCL Genomics (UCLG). Briefly, RNA integrity was confirmed using the Agilent 4200 TapeStation System (RRID:SCR_018435). Samples were processed in batches using either the TruSeq Stranded mRNA Library prep kit (Illumina, 20020595) or the KAPA mRNA HyperPrep Kit (Roche p/n KK8580) according to manufacturer’s instructions. Samples were sequenced on the Illumina NovaSeq 6000 Sequencing System (RRID:SCR_016387) at 300 pM, using a 101 bp paired read run with corresponding 8 bp dual sample index and 8 bp unique molecular index reads. Run data were demultiplexed and converted to fastq files using Illumina’s BCL Convert Software v3.75.

Transcript abundance was estimated from fastq files of paired-reads generated by UCLG. Reads were mapped to the human genome using STAR aligner (RRID:SCR_004463) [44] (Ref genome: Ensembl GRCh38) and summarized with featureCounts (RRID:SCR_012919). All sequence and annotation data were obtained from the Illumina iGenomes repository. Quality control was conducted on the bulk RNA-seq read count-table obtained from featureCounts to ensure data quality. Samples with fewer than 5 million reads were excluded, as these were deemed insufficient for reliable analysis. Detailed RNA sequencing data analysis workflow describing quality control, alignment, and generation of raw transcript counts can be found in the following GitHub repository: https://github.com/WedderburnLab/RNAseq-Pipeline. Read counts were kit-corrected with CombatSeq [45] prior to differential gene expression analysis.

### Transcriptional data analysis

Statistical analysis and visualization of transcriptional data were performed using R software (RRID:SCR_001905) and Bioconductor (RRID:SCR_006442) packages [46] including DESeq2 (RRID:SCR_015687) [47, 48] and EnhancedVolcano (RRID:SCR_018931) [49]. Normalization and differential analysis were conducted according to the DESeq2 model and package, controlling for sex, age, and batch. The *P*-values obtained were corrected for multiple testing using the Benjamini–Hochberg method. An adjusted *P*-value of less than 0.05 was used to identify differentially expressed genes (DEG). DEG were visualized in volcano plots generated using the EnhancedVolcano package.

### Pathway and gene set enrichment analysis

Pathway enrichment analysis was performed using the Metascape gene annotation and analysis resource (RRID:SCR_016620) [50] and GO biological process (GO BP) ontology catalogue. Upregulated DEG and downregulated DEG gene lists were analysed separately with significance determined by a *P*-value cut-off of 0.01 and a minimum enrichment ratio (ER) of 1.5. As a complementary approach, gene set enrichment analysis (GSEA) of all genes analysed based on differential expression rank was performed using GSEA 4.2.2 software (RRID:SCR_003199) [51] with the GO BP v2023.1 Molecular Signatures Database (RRID:SCR_016863) gene set collection [52]. Statistical significance of the normalized enrichment scores (NES) was determined by phenotype permutation. Gene set enrichment was deemed significant at false discovery rate (FDR) q value < 0.25.

### Interferon and mitochondrial gene scores

Type I IFN and mitochondrial gene scores were calculated as described [42]. Transcript per million (TPM) counts of 15 known interferon-stimulated genes *(IFI27, IFI44, IFI44L, IFI6, IFIT1, IFIT3, IRF7, ISG15, LY6E, MX1, OAS1, RSAD2, SIGLEC1, STAT1, USP18)* [53] 13 polypeptide-encoding genes within the mitochondrial genome (*MT-ATP6, MT-ATP8, MT-CO1, MT-CO2, MT-CO3, MT-CYB, MT-ND1, MT-ND2, MT-ND3, MT-ND4, MT-ND4L, MT-ND5, MT-ND6*) [54] were normalized to the highest value for each gene and summed to produce the type I IFN score and mitochondrial gene score for each individual.

### Serum measurements

#### LEGENDplex^TM^ assay

A commercial 13-plex flow cytometry bead assay (CD8/NK LEGENDplex^TM^, BioLegend, 740267) was used to quantify the expression of the following analytes: IL-2, IL-4, IL-10, IL-6, IL-17A, TNF-α, sFas, sFasL, IFN-γ, granzyme A, granzyme B, perforin, and granulysin, in patient and HC serum. The assay was performed according to manufacturer’s instructions and run on a BD FACSVerse flow cytometer. Raw flow cytometry data were gated and processed using BioLegend’s LEGENDplex^TM^ Data Analysis Software v8.0. Only TNF-α, sFas, sFasL, IFN-γ and perforin levels are shown. All of the analytes measured in HC fell within the expected range for healthy serum, with the exception of two HC with unusually high IFN-γ and TNF-α levels. The same two individuals who had high IFN-γ levels also had the highest serum TNF-α levels.

#### Serum metabolites

Measures of non-lipid serum biomarkers were acquired in 32 JSLE patients and 42 HC by nuclear magnetic resonance (NMR) spectroscopy using a well-established platform conducted by Nightingale Health Ltd [55]. Twenty-three measures were collected, including: amino acids (alanine, glutamine, glycine, histidine, total BCAA (branched-chain amino acids: leucine + isoleucine + valine), isoleucine, leucine, valine, phenylalanine, tyrosine), glycolysis-related metabolites (glucose, lactate, pyruvate, citrate, glycerol), ketone bodies (3-hydroxybutyrate, acetate, acetoacetate, acetone, ratio of 3-hydroxybutyrate to acetoacetate), fluid balance (creatinine, albumin), and inflammation (glycoprotein acetyls). Ratio of 3-hydroxybutyrate to acetoacetate was not specifically measured by the platform, however it was calculated from 3-hydroxybutyrate and acetoacetate measures and included in the analysis as this ratio has been shown to reflect the mitochondrial oxido-reduction state [56].

### Statistical analysis

Data were analysed using R software version 4.2.2 (RRID:SCR_001905) and plots were produced using the ggplot2 package (RRID:SCR_014601) [57]. Population distributions were visualized using density plots and qq-plots. Formal Shapiro–Wilk normality testing was also performed to assess normality. In large data sets with *n* > 90 if data points fell vastly outside the distribution robust regression and outlier (ROUT) analysis was performed in GraphPad Prism 10 (RRID:SCR_002798). Unpaired or paired two-sided Mann–Whitney U tests or Student’s t-tests were applied to test differences between two groups as appropriate depending on the data distribution. Kruskal–Wallis test with Dunn’s test post hoc testing was applied when comparing more than two groups. To correlate clinical and demographic parameters Spearman or Pearson correlation was used in accordance with the distribution of the data.

Multiple linear regression analysis of metabolomic data were performed using the ggforestplot package in R to assess which metabolites were significantly different in JSLE when sex, age, and ethnicity were taken into account. Metabolite measures were log(1 + *x*) transformed and scaled prior to regression. Regression results were visualized on a forest plot with FDR adjusted *P*-value < 0.05 used to determine statistical significance.

## Results

### Reduction in CD8^+^ T cell cytotoxic capacity in JSLE is an underlying feature of the disease

**Figure 1.**
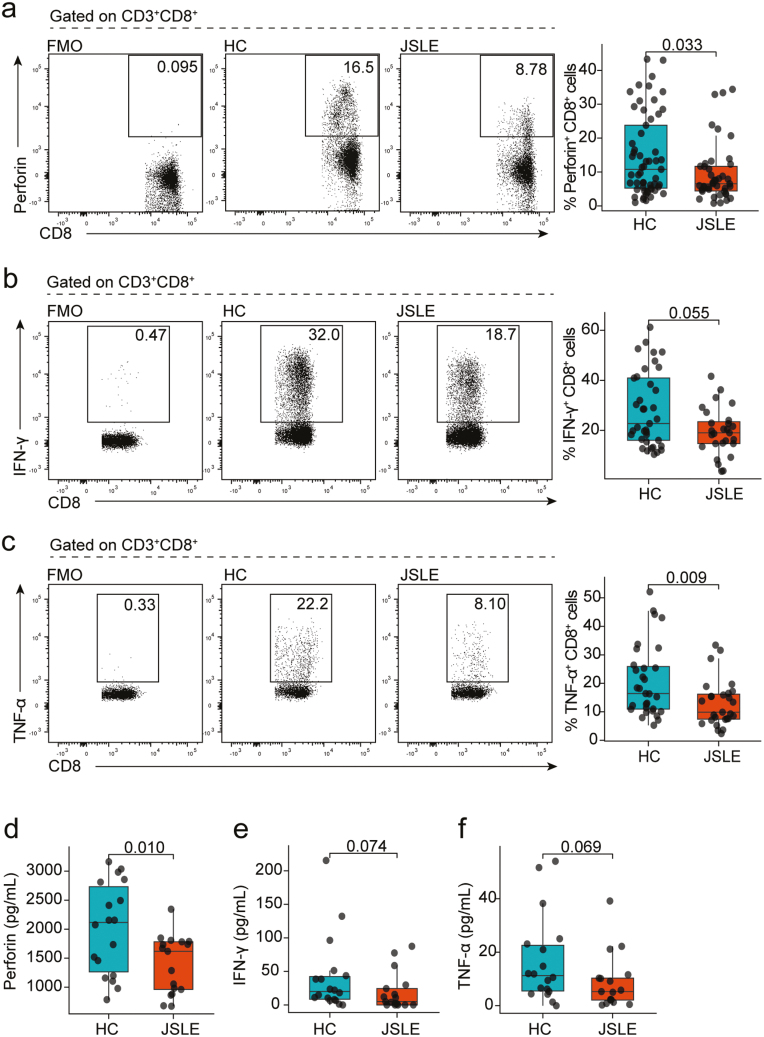
Reduction in cytotoxic CD8^+^ T cell capacity in JSLE. Representative flow plots and box plots showing in *ex vivo* PBMCs the percentage of CD8^+^ T cells (a) expressing perforin (HC *n* = 57, JSLE *n* = 42) in HC and JSLE patients. Representative flow plots and box plots showing percentage of CD8^+^ T cells expressing (b) IFN-γ (HC *n* = 37, JSLE *n* = 27), and (c) TNF-α (HC *n* = 31, JSLE *n* = 27) in PBMCs cultured with PMA/ionomycin in the presence of brefeldin and monensin for 4 h. Boxplots quantifying levels of (d) perforin, (e) IFN-γ, and (f) TNF-α in serum of HC (*n* = 18) and JSLE patients (*n* = 17). Analytes were measured using a flow cytometry-based bead multiplex assay. All boxplots show median ± IQR. Flow plots show percentage of cells within each gate. *P*-values calculated using unpaired Mann–Whitney U test (a–c, e–f) or unpaired t-test as appropriate (d). HC: healthy controls; IFN: interferon; IQR: interquartile range; JSLE: juvenile systemic lupus erythematosus; PBMCs: peripheral blood mononuclear cells; PMA: phorbol 12-myristate 13-acetate; TNF: tumour necrosis factor.

Previously published research has demonstrated that the cytotoxic capacity of CD8^+^ T cells is altered in adult-onset SLE (reviewed in [7]). To characterize whether similar alterations are observed in juvenile-onset disease, the frequency of CD8^+^ T cell populations expressing cytotoxic markers and effector cytokines in JSLE patients and age-matched HC were assessed (see Table 1). This demonstrated that there was a significant reduction in perforin^+^ CD8^+^ T cells in JSLE compared to HC (*P* = 0.033, Fig. 1a). In addition, there was a strong trend for a reduction in frequencies of IFN-γ^+^ CD8^+^ T cells in JSLE (Fig. 1b, *P* = 0.055) and a significant reduction in TNF-α^+^ CD8^+^ T cells in JSLE (Fig. 1c, *P* = 0.009) compared to HC. In keeping with the observed reduction in perforin^+^ CD8^+^ T cells, there was also trend for a reduction in frequencies of CD8^+^ T cells expressing the degranulation marker CD107a in JSLE compared to HC (*P* = 0.068, Fig. S1a). These data were mirrored by a significant reduction in perforin (*P* = 0.01) and a trend for a reduction in IFN-γ (*P* = 0.074) and TNF-α (*P* = 0.069) in the serum of JSLE compared to HC (Fig. 1d–f). Of note, there were no significant differences in total CD8^+^ T cells, granzyme A^+^, granzyme B^+^, or granulysin^+^ CD8^+^ T cells and no difference in soluble Fas (sFas) or soluble FasL (sFasL) in serum between JSLE versus HC (Fig. S1b–g).

As JSLE is a highly heterogeneous disease and parameters such as clinical and serological disease activity, organ involvement, and medication use influence immune phenotype, we next wanted to confirm that the observed reduction in perforin^+^, IFN-γ^+^, and TNF-α^+^ CD8^+^ T cell frequencies was not due to the impact of numerous clinical variables. No significant differences in CD8^+^ T cell subpopulations were observed between JSLE patient groups stratified by SLEDAI-2K (Fig. S2a–c), the global BILAG-2004 index score (Fig. S3a–c), any of the nine organ domains evaluated in the BILAG (Fig. S3d) or by different medication usage including mycophenolate mofetil (MMF) (Fig. S4a–c), azathioprine (AZA) (Fig. S4d–g), hydroxychloroquine (HCQ) (Fig. S4g–i), or oral prednisolone (Fig. S4j–l). In addition, frequencies of CD8^+^perforin^+^ and CD8^+^TNF-α^+^ in JSLE did not correlate with any of the standard tested haematological or serological parameters including leukocyte counts, lymphocyte counts, complement 3 (C3) or anti-dsDNA (Fig. S5). However, there was a positive correlation in JSLE patients between frequencies of IFN-γ producing CD8^+^ T cells and lymphocyte counts (r = 0.44, *P* = 0.02) (Fig. S5e). In addition, perforin^+^, IFN-γ^+^, and TNF-α^+^ CD8^+^ T cell population frequencies did not correlate significantly with age (Fig. S6a–c) nor were significant differences in these populations observed between self-reported sex in HC or JSLE (Fig. S7a–c). Thus, the reduction in CD8^+^ T cell cytotoxic effector cells between JSLE versus HC appeared to be an underlying feature of disease rather than due to the influence of various clinical or demographic variables.

### CD8^+^ T cell cytotoxic populations in JSLE are functional and not exhausted or senescent

**Figure 2.**
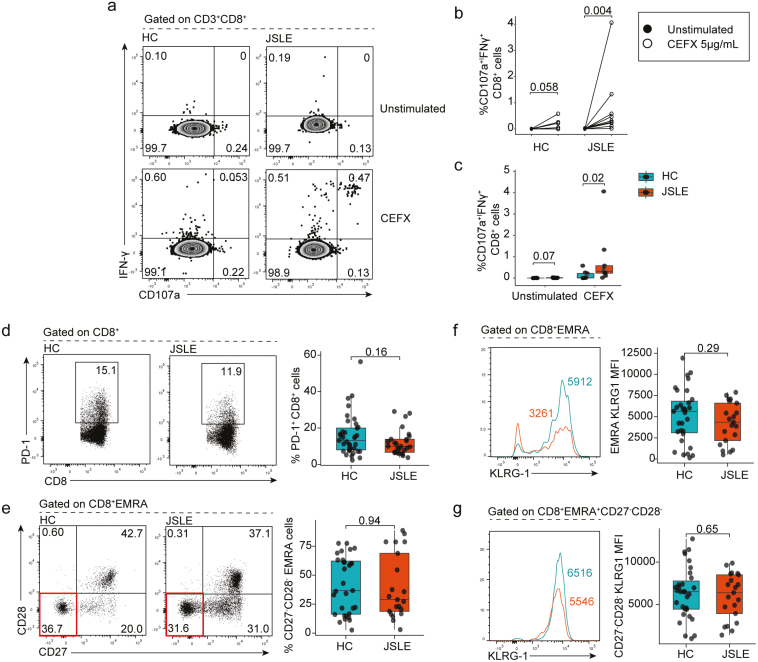
JSLE CD8^+^ T cells are functional and do not exhibit an exhausted or senescent phenotype. PBMCs from JSLE patients and HC were cultured in the presence or absence of CEFX (a pool of synthetic peptides of known MHC-I restricted epitopes) for 6 h. (a) Representative flow plots and (b) graph quantifying the percentage of CD107a^+^IFN-γ^+^ CD8^+^ T cells in unstimulated (DMSO only) and CEFX peptide treated HC (*n* = 9) and JSLE (*n* = 9) PBMCs are shown. (c) Boxplots quantifying differences in percentage of CD107a^+^IFN-γ^+^ CD8^+^ T cells in HC vs JSLE across all experimental conditions. Representative flow diagrams and boxplots showing (d) frequencies of PD-1 expressing CD8^+^ T cells in JSLE (*n* = 27) and HC (*n* = 37) in PBMCs stimulated with PMA/ionomycin and (e) *ex vivo* frequencies of terminally differentiated CD27^−^CD28^−^ EMRA cells (highlighted in red in bottom flow panel) in JSLE (*n* = 21) and HC (*n* = 31). (f) KLRG1 expression in EMRA and CD27^−^CD28^−^ EMRA cells JSLE (*n* = 21) and HC (*n* = 31). Line plots show KLRG1 MFI in indicated subsets. Numbers in gates and quadrants in flow plots indicate percentage of cells. All boxplots show median ± IQR. *P*-values calculated using unpaired Mann–Whitney U test (c, d, e), paired Mann–Whitney U test (b) or unpaired t-test (f, g) as appropriate to data distribution. DMSO: dimethyl sulfoxide; EMRA: effector memory cells re-expressing CD45RA; HC: healthy controls; IFN: interferon; IQR: interquartile range; JSLE: juvenile systemic lupus erythematosus; KLRG1: killer cell lectin-like receptor G1; MFI: mean fluorescence intensity; MHC-I: major histocompatibility complex type I; PBMCs: peripheral blood mononuclear cells; PD-1: programmed cell death protein-1; PMA: phorbol 12-myristate 13-acetate.

To interrogate this in more detail and to understand if differences in cytotoxic potential in JSLE resulted in inability of CD8^+^ T cells to respond to antigenic stimulation, we stimulated PBMCs from JSLE patients and controls with a combination of viral peptides containing MHC-I-restricted epitopes. This demonstrated that CD8^+^ T cells from JSLE patients were functional and in fact exhibited enhanced degranulation responses upon stimulation as evidenced by higher proportions of CD107a^+^IFN-γ^+^ CD8^+^ T cells compared to HC (*P* = 0.02) (Fig. 2a–c). In line with these findings, we found no difference in the percentage of exhausted PD-1^+^CD8^+^ T cells in JSLE and HC (Fig. 2d), or senescent CD27^−^CD28^−^ EMRA (effector memory cells re-expressing CD45RA) populations (Fig. 2e) between JSLE and HC. There were also no significant differences observed in killer cell lectin-like receptor G1 (KLRG1) expression—which is expressed on cells that have lost their proliferative capacity [58]—on EMRA or EMRA CD27^−^CD28^−^ between JSLE and HC (Fig. 2f–g). Thus, JSLE CD8^+^ T cells are functional and not exhausted or senescent suggesting that other mechanisms may be responsible for reduction in CD8^+^ T cell cytotoxic capacity in JSLE.

### Transcriptomics reveals strong type I IFN signatures and mitochondrial defects in JSLE CD8^+^ T cells

**Figure 3.**
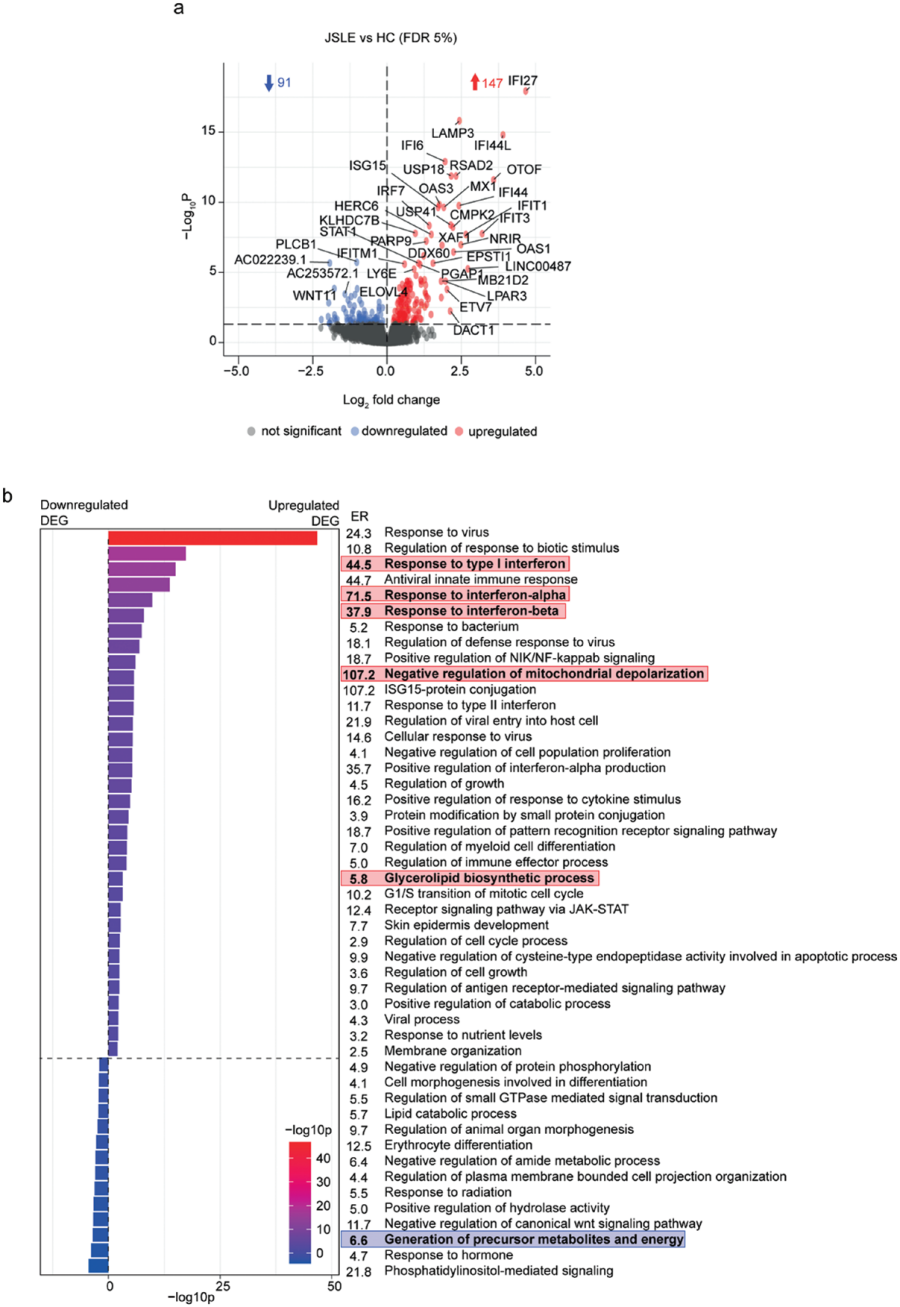
Transcriptomic analysis reveals upregulation of IFN-α responses and potential metabolic and mitochondrial disturbances in CD8^+^ T cells in JSLE. (a) Volcano plot showing differences in gene expression from RNA sequencing of CD8^+^ T cells from JSLE (*n* = 26) vs HC (*n* = 29). Blue and red points represent statistically significant differentially expressed genes below the FDR adjusted *P*-value threshold of 0.05. Blue and red arrows indicate number of statistically significant downregulated and upregulated genes, respectively. (b) Bar plot showing −log10p values and enrichment ratios (ER) of summary enriched pathway GO BP ontology terms in CD8^+^ T cells in JSLE vs HC using the 147 significantly upregulated and 91 significantly downregulated genes (FDR adjusted *P* < 0.05). Statistical significance of enrichment was determined using a *P*-value cut-off of 0.01 and a minimum enrichment score of 1.5. Terms highlighted in red and blue represent pathways of potential interest, derived from upregulated (red) and downregulated (blue) genes in JSLE vs HC. DEG: differentially expressed genes; ER: enrichment ratio; FDR: false discovery rate; GO BP: gene ontology biological process; HC: healthy controls; ISG-15: interferon-stimulated gene 15; JAK-STAT: Janus kinase/signal transducers and activators of transcription; JSLE: juvenile systemic lupus erythematosus; NF-kappaB: nuclear factor kappa-light-chain-enhancer of activated B cells; NIK: NF-kappaB-inducing kinase; RNA: ribonucleic acid.

To investigate the potential processes underlying the reduction in cytotoxic CD8^+^ T cell capacity in JSLE, we performed RNA sequencing transcriptomic analysis of sorted CD8^+^ T cells from age-, sex-, and ethnicity-matched HC (*n* = 29) and JSLE patients (*n* = 26) (demographics in Tables S1 and S2), which had similar disease characteristics to samples included in our phenotyping studies (Table S3). Transcriptomic analysis identified 147 upregulated and 91 downregulated transcripts (FDR adjusted *P* < 0.05) in JSLE compared to HC (Fig. 3a) with the top 10 upregulated and downregulated genes alongside their functions shown in Table S4. Pathway analysis of differentially expressed genes (DEG) in JSLE patients vs HC revealed several pathways of interest, including statistically significant Gene Ontology Biological Process (GO BP) ontology terms related to upregulation of ‘response to type I IFN’ (*P* = 9.8 × 10^−16^), which is a widely reported feature of multiple immune cell populations in SLE and JSLE [20], ‘negative regulation of mitochondrial depolarization’ (*P* = 2.0 × 10^−6^), ‘glycerolipid biosynthetic process’ (*P* = 0.0006), and a downregulation of the pathway ‘generation of precursor metabolites and energy’ (*P* = 0.0003) (Fig. 3b). Pathway enrichment analysis results were complemented by gene set enrichment analyses (GSEA) demonstrating that IFN-α response was again the most prominent pathway over-represented in JSLE (Fig. S8). Similarly to pathway enrichment analysis, GSEA also highlighted changes in mitochondrial function (mitochondrial RNA metabolic process and mitochondrial transcription).

### Abnormal mitochondrial morphology and distinct non-lipid serum metabolomic signature in JSLE

**Figure 4.**
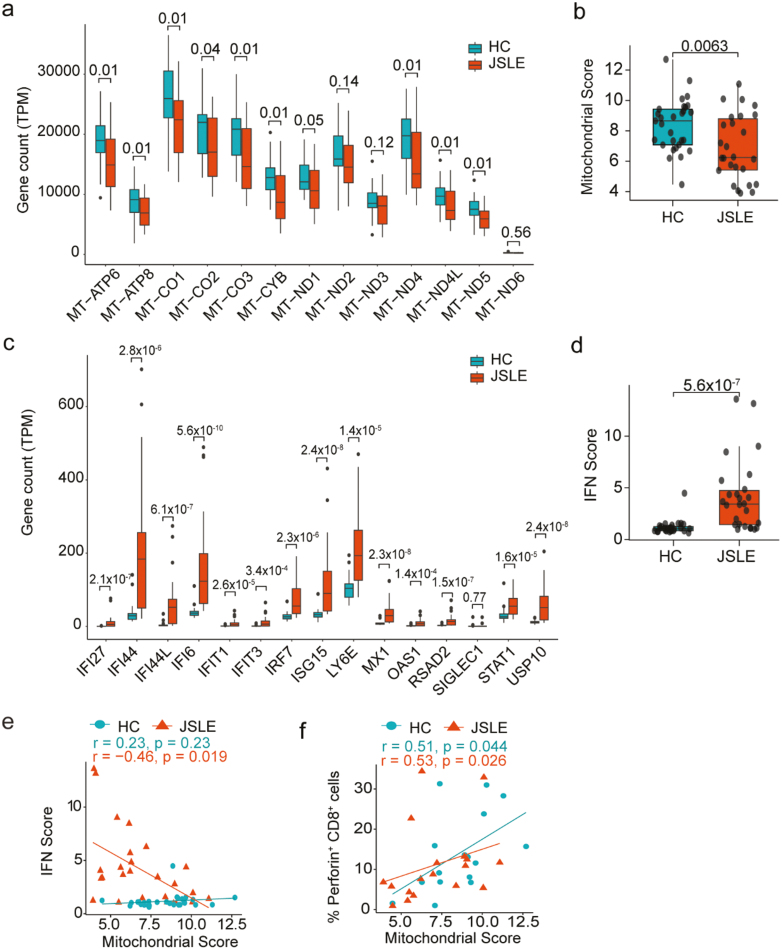
Type I IFN gene scores are increased and correlate negatively with mitochondrial gene expression in CD8^+^ T cells in JSLE. (a) Boxplots displaying transcript per million (TPM) gene counts of 13 mitochondrially expressed genes in HC (*n* = 29) and JSLE (*n* = 26) CD8^+^ T cells from RNA-seq. FDR corrected *P*-values are shown. (b) Boxplot quantifying mitochondrial score, calculated by taking expression of all genes in (a) into account, in JSLE (*n* = 26) vs HC (*n* = 29). (c) Boxplots displaying transcript per million (TPM) gene counts of 15 type I IFN-stimulated genes in HC (*n* = 29) and JSLE (*n* = 26) CD8^+^ T cells from RNA-seq. FDR corrected *P*-values are shown. (d) Box plot of interferon score in HC (*n* = 29) and JSLE (*n* = 26) samples. All boxplots show median ± IQR. *P*-values calculated using unpaired t-test (a, b) or Mann–Whitney U test (a, c, d) as appropriate to data distribution. Scatter plots showing (e) correlations between type I IFN score and mitochondrial score (HC: *n* = 29, JSLE: *n* = 26) and (f) correlations between mitochondrial score and frequencies of perforin-expressing CD8^+^ T cells (HC *n* = 16, JSLE *n* = 18). Spearman’s rho correlation coefficients and the associated *P*-values are shown (e, f). FDR: false discovery rate; HC: healthy controls; IFN: interferon; IQR: interquartile range; JSLE: juvenile systemic lupus erythematosus; RNA-seq: RNA sequencing; TPM: transcript per million.

To further interrogate the connection between type I IFN, mitochondrial dysfunction and altered CD8^+^ T cell function, we assessed transcript levels for the expression of the 13 polypeptide-encoding genes within the mitochondrial genome [54] and 15 known interferon-stimulated genes used to derive the interferon score [53] within CD8^+^ T cells. This showed that 12 of the 13 mitochondrially encoded genes exhibited reduced expression in CD8^+^ T cells in our JSLE cohort (Fig. 4a), resulting in an overall reduction in mitochondrial gene score (*P* = 0.0063, Fig. 4b) and 14 of the 15 IFN genes were significantly upregulated in our JSLE cohort (Fig. 4c) leading to increased interferon scores in JSLE compared to HC (Fig. 4d). Interestingly, we found that reduced mitochondrial gene expression was associated with both increased type I IFN gene expression in JSLE (Fig. 4e, r = −0.46, *P* = 0.019) and reduction in the frequency of CD8^+^perforin^+^ T cells in JSLE and HC (*P* = 0.026, *P* = 0.044, Fig. 4f) suggesting a direct link between these observations. Of note, there were no differences in transcript expression of cytotoxic molecules or effector cytokines (*GZMB*, *GZMA*, *GNLY*, *PRF*1, *TNF*, *IFNG*) in JSLE vs HC (data not shown).

**Figure 5.**
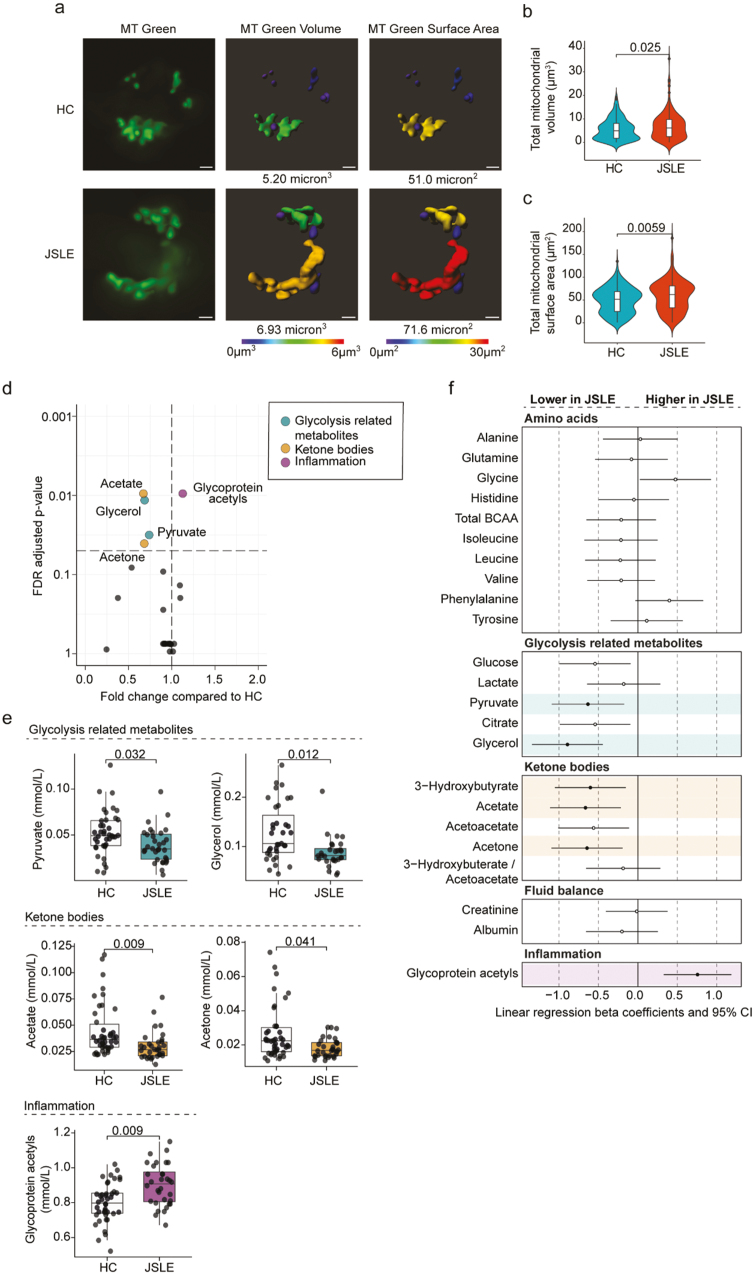
Alterations in CD8^+^ T cell mitochondrial morphology and non-lipid serum metabolome in JSLE. (a) Representative images of CD8^+^ T cells from JSLE patients (*n* = 3) and HC (*n* = 4) stained with MitoTracker (MT) Green and 3D modelling images showing mitochondrial volume and surface area. White bars represent 1µm. (b) Violin plot showing the distribution of total mitochondrial volume (µm^3^) per cell in JSLE CD8^+^ T cells (*n* = 3) compared with HC (*n* = 4). (c) Violin plot showing the distribution of total mitochondrial surface area (µm^2^) from all JSLE CD8^+^ T cells (*n* = 3) compared with HC (*n* = 4). *P*-values for HC vs JSLE comparisons calculated using unpaired Mann–Whitney U test (b, c). (d) Volcano plot showing fold changes and FDR adjusted *P*-values obtained from individual Mann–Whitney tests of non-lipid metabolites in JSLE compared to HC. The significance threshold (FDR *P*-value < 0.05) is indicated by the horizontal dashed line. Significantly altered metabolites are highlighted in colour according to metabolite group. (e) Boxplots plotting serum levels of metabolites altered in JSLE showing median ± IQR. FDR adjusted *P*-values calculated using unpaired Mann–Whitney U test are shown. (f) Forest plot summarizing results of linear regression analysis for each metabolite (log transformed and scaled), showing which metabolites are significantly altered in JSLE when accounting for sex, age, and ethnicity. Regression results highlighted in colour by metabolite group and filled circles indicate significantly altered metabolites (FDR *P*-value <0.05). FDR: false discovery rate; HC: healthy controls; IQR: interquartile range; JSLE: juvenile lupus erythematosus; MT: MitoTracker.

To assess whether altered mitochondrial gene expression led to altered mitochondrial biology in CD8^+^ T cells and more globally to metabolic alterations in JSLE, we carried out two complementary analyses. First, we assessed mitochondrial morphology in JSLE CD8^+^ T cells compared to controls, which demonstrated that JSLE CD8^+^ T cells had increased mitochondrial volume (Fig. 5a and b) and surface area (Fig. 5a and c) compared to HC (*P* = 0.025 and *P* = 0.0058, respectively). Next, as both type I IFN and altered mitochondrial function have been linked with global changes in metabolic processes and our transcriptional analysis had highlighted multiple pathways associated with changes in cellular metabolism [59, 60], we analysed the non-lipid serum metabolomic profile of 32 JSLE patients and 42 HC. Mann Whitney testing with FDR correction revealed that levels of 4 metabolites (pyruvate, glycerol, acetate and acetone) were significantly decreased in JSLE, while glycoprotein acetyls (GlycA) were significantly increased (Fig. 5d and e). Notably, we found that these differences remained after assessment of the metabolome by linear regression analysis to account for sex, age and ethnicity, as well as additionally identifying reduction in 3-hydroxybutyrate in JSLE (Fig. 5f). The changes in serum metabolites observed in JSLE, especially the reduction in levels of ketone bodies (3-hydroxybutyrate, acetate and acetone), which are produced by fatty acid oxidation in the mitochondria [61], and the reduction in the glycolysis-related metabolites glycerol and pyruvate, provide further evidence of altered mitochondrial and metabolic function in JSLE.

### IFN-α-induced apoptosis of effector memory CD8^+^ T cells in JSLE

**Figure 6.**
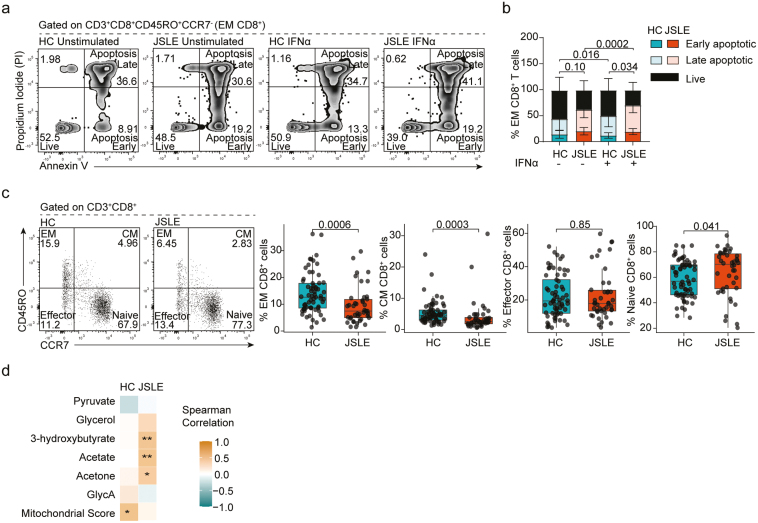
EM CD8^+^ T cells in JSLE are susceptible to IFN-α-induced apoptosis. (a) Representative flow plots showing Annexin V and PI staining. (b) Stacked bar plot quantifying frequencies of early apoptotic, late apoptotic, and live EM CD8^+^ T cells in HC (*n* = 9) and JSLE patients (*n* = 10) in PBMCs with and without stimulation with IFN-α for 48 h. Bar plot displays means ± SD. *P*-values comparing total apoptotic cells (early apoptotic + late apoptotic) were calculated using unpaired t-test in HC vs JSLE comparisons and paired t-test for unstimulated vs IFN-α stimulated comparisons. (d) Representative flow plots and boxplots showing percentage of CM, EM, effector, and naïve CD8^+^ T cell populations in HC (*n* = 65) and JSLE (*n* = 42). Numbers in all flow plot quadrants indicate percentage of cells. All boxplots show median ± IQR. *P*-values for HC vs JSLE comparisons calculated using Mann–Whitney U test. (d) Correlation matrix showing strength of correlation between frequency of EM CD8^+^ T cells and differentially expressed metabolites and mitochondrial score in HC and JSLE. Spearman correlations are shown. *P < 0.05, **P < 0.01. EM: effector memory; CM: central memory; GlycA: glycoprotein acetyls; HC: healthy controls; IFN: interferon; IQR: interquartile range; JSLE: juvenile systemic lupus erythematosus; PBMCs: peripheral blood mononuclear cells; PI: propidium iodide; SD: standard deviation.

To understand how altered IFN biology and changes in mitochondrial function are linked to altered cytotoxic responses, we assessed the direct impact of IFN-α on CD8^+^ T cell biology and more specifically CD8^+^ T cell apoptosis *in vitro*. Compromised mitochondrial integrity has been proposed to enhance expression of type I IFN-stimulated genes during sterile inflammation (e.g. autoimmune conditions) and as a trigger of apoptosis or cell death [62]. This demonstrated that while there was no difference in IFN-α-induced apoptosis of total CD8^+^ T cells (Fig. S9), effector memory (EM) CD8^+^ T cells in JSLE were more susceptible to apoptosis in the presence of IFN-α than their HC counterparts (*P* = 0.034, Fig. 6a and b). Previous published research has demonstrated that within the CD8^+^ T cell compartment, cytotoxic mediators and cytokines are predominantly produced by memory and effector cells [63, 64] and selective type I IFN-induced apoptosis of pre-existing memory CD8^+^ T cells during infection has been documented [65, 66]. Thus, to confirm the relevance of our *in vitro* experiments to our observations *ex vivo*, we evaluated the proportion of CD8^+^ T cell subsets within PBMCs and found that EM CD45RO^+^CCR7^−^ (*P* = 0.0006) and central memory (CM) CD45RO^+^CCR7^+^ (*P* = 0.0003) CD8^+^ T cell subsets were reduced in JSLE, while the proportion of naïve CD45RO^-^CCR7^+^ CD8^+^ T cells was increased (*P* = 0.041, Fig. 6c). When split by CD8^+^ T cell subset, in agreement with published data, EM CD45RO^+^CCR7^−^ CD8^+^ T cells were the highest overall producers of perforin, IFN-γ, or TNF-α of any CD8^+^ T cells in both JSLE patients and HC. In addition, there were no longer any significant differences in expression of perforin, IFN-γ, or TNF-α between JSLE patients and HC demonstrating that the reduction in cytotoxic cell frequency in total CD8^+^ T cells was driven by the deficiency in EM CD45RO^+^CCR7^−^ CD8^+^ T cells (Fig. S10).

Finally, to understand the association of reduced EM CD45RO^+^CCR7^−^ CD8^+^ T cells with mitochondrial abnormalities including metabolic disturbances, we correlated frequencies of EM CD8^+^ T cells in JSLE and HC with the mitochondrial gene score in matched total CD8^+^ T cells and the levels of non-lipid metabolites differentially expressed in JSLE serum (Fig. 6d). There was a significant positive correlation between EM CD8^+^ T cell frequency and the mitochondrial gene score in total CD8^+^ T cells in HC, which was lost in JSLE. In addition, we found that while there was no association between EM CD8^+^ T cell frequency and glycolysis-associated metabolites (pyruvate, glycerol) ketone bodies (3-hydroxybutyrate, acetate or acetone) and GlycA in HC serum, there was a positive correlation between the EM CD8^+^ T cell frequency and level of ketone bodies in JSLE serum (Fig. S11).

## Discussion

To date, there are no studies that have carried out an in-depth analysis of CD8^+^ T cell cytotoxic profiles in JSLE and findings in adult-onset SLE remain inconclusive (reviewed in [7]). In our study, we demonstrate in a representative JSLE cohort (JSLE is up to 10 times rarer than adult-onset SLE) that there is a reduction in cytotoxic CD8^+^ T cells within the peripheral blood of JSLE patients compared to controls, irrespective of disease activity or treatment, which is due to a numerical defect in EM CD8^+^ T cells. We also demonstrate that the transcriptome of JSLE CD8^+^ T cells is distinguished from HC by a dysregulation in pathways associated with both type I IFN signature and mitochondrial biology, with IFN-α specifically increasing JSLE EM CD8^+^ T cell apoptosis *in vitro*. Based on these data, we hypothesize that the numerical defect in EM CD8^+^T cells, and therefore a reduction in total cytotoxic CD8^+^ T cells, is caused by mitochondrial dysfunction and increased type I IFN signalling, leading to increased apoptosis.

Our study adds to the growing evidence that CD8^+^ T cell biology is altered in both adult-onset and juvenile SLE. Significant discrepancies exist between studies which may be explained by the inherent disease heterogeneity, as well as more specific genetic, environmental, and socio-economic factors which are unique to every cohort. For example, increased total CD8^+^ T cell frequencies have been reported in JSLE [35, 67], with one study associating these elevated levels with milder disease and the absence of vasculitis and lupus nephritis [67]. Reduction in frequencies of CM, EM, and EMRA CD8^+^ T cells in JSLE patients compared to HC were also reported [35], with other studies finding no differences in CD8^+^ T cell populations between JSLE patients and HC [68, 69]. In this study, to address this heterogeneity and investigate CD8^+^ T cell immunophenotype in a real-life cohort of well-characterized JSLE patients (majority with well-controlled or low disease activity), we assessed the levels of multiple cytotoxic markers in JSLE patients versus age- and sex-matched controls and identified that there was a marked reduction in CD8^+^ T cells expressing the effector cytokines IFN-γ and TNF-α and the cytotoxic mediator perforin. Importantly, these reductions were evident regardless of whether the patients were stratified based on clinical disease activity, drug treatment, or organ involvement, suggesting that the disease itself rather than specific JSLE characteristics within this cohort were driving these observed differences in the peripheral blood.

Although, patients and controls were matched for age, sex, and ethnicity within the study, these factors can vary significantly between published studies and may contribute to some of the differences between our study and published literature. Of note, a recent report of transcriptomic analysis of single-cell RNA-seq data from PBMCs in childhood SLE by Nehar-Belaid et al, identified fractions of CD8^+^ T cells in JSLE expressing a strong cytotoxic program [20]. Although the exact reasons for differences in the phenotypes observed in the present study and this transcriptional profiling study remain unclear, the majority of the patients in the transcriptomic study were of Hispanic (42%) or African American (36%) ethnicity, while these ethnicities are not at all represented in our cohort. Current renal disease (24% vs 4% in this cohort) and oral steroid use (60% vs 23% in this cohort) are also much more prevalent in their cohort. In addition, it is as yet unclear why this strongly cytotoxic fraction was only present in some patients and post-transcriptional regulatory processes must not be overlooked, as in eukaryotes only about 40% of the variation in protein abundance can be explained by quantifying mRNA transcripts [70]. The lack of appreciable differences in transcriptomic profiles seen between paediatric and adult lupus populations despite existing evidence that juvenile lupus is a more aggressive disease with widespread organ involvement, highlights the fact that transcriptomic analysis is not the full story, but may nevertheless offer valuable clues in dissecting the pathogenesis of the disease.

Although there were no differences in frequencies of total CD8^+^ T cells in this cohort of juvenile patients, the proportions of memory subsets (both EM and CM) were significantly diminished. Since cytotoxic mediators and cytokines are expressed primarily by memory and effector cells, the reduction in total CD8^+^ T cell cytotoxic populations may be partially due to the reduction in memory cells in JSLE. There could be a number of reasons for the observed reduction in memory cells, including migration of these cells out of peripheral blood into tissues or increased cell death. In fact, we observed that EM cells from JSLE patients exhibited enhanced apoptotic cell death, in particular in the presence of IFN-α, indicating that enhanced apoptosis may, at least in part, explain the reduction in CD8^+^ T cell cytotoxic capacity in JSLE. Memory CD8^+^ T cells in mice have been shown to express higher levels of IFNR1 (interferon receptor 1) than naïve cells [71] and selective IFN-induced apoptosis of pre-existing memory CD8^+^ T cells during infection has been documented [65, 66], raising the possibility that this mechanism of IFN-α induced memory CD8^+^ T cell death may be impacting CD8^+^ T cell population dynamics in JSLE. Importantly, these observations complement our recent findings demonstrating that cytotoxic natural killer (NK) cells are also reduced in JSLE, and that this is linked to IFN-α-induced apoptosis [72]. Although our previous findings demonstrate that the reduction in NK cell frequency in JSLE is linked to disease activity, while cytotoxic CD8^+^ T cell frequency is reduced regardless of disease activity, these data demonstrate that dysregulated type I IFN signalling leads to a global reduction in cytotoxic capacity in JSLE which could have important implications for autoantigen spreading due to reduced clearance of apoptotic cells.

Additionally, the diminished frequency of memory cells in peripheral blood may be due to migration of memory cells into tissues. In support of this, others have shown the presence of CD8^+^ T cell infiltrates in lupus nephritis kidney biopsies [73], and CD8^+^ T cells have been found in urine of patients with active lupus nephritis [74], and in skin biopsies in cutaneous lupus [75]. These CD8^+^ T cells have been shown to predominantly have an EM phenotype [76, 77]. Furthermore, transcriptomic single cell analysis has revealed the presence of subsets of tissue resident memory CD8^+^ T cells and cytotoxic CD8^+^ T cells expressing perforin, granzyme B and granulysin in renal tissue from patients with lupus nephritis [78]. To address this, it would be informative to enumerate memory CD8^+^ T cells in biopsies (e.g. from the skin, kidney) in patients with JSLE. Unfortunately, due to difficulties in obtaining these samples that was not possible during this study.

Reduction of memory cells in JSLE due to increased apoptosis and migration are two possible explanations for the decrease in CD8^+^ T cell populations expressing cytotoxic mediators and cytokines in JSLE. An additional possibility is that low levels of persistent autoantigen in JSLE may lead to exhaustion and gradual loss of effector function and cytokine secretion. However, we observed no differences in expression of the exhaustion marker PD-1 in CD8^+^ T cells, and JSLE CD8^+^ T cells exhibited preserved cytotoxic responses upon *in vitro* stimulation with antigen compared to HC cells, making exhaustion unlikely. The preservation of antigen-specific responses observed in JSLE is somewhat surprising given the reduction in the production of CD8^+^ T cell cytotoxic effector molecules noted under ionophore stimulation. Considering that there are no differences in frequencies of EM CD8^+^ T cells producing IFN-γ and TNF-α between JSLE and HC upon ionophore stimulation, it appears that the reduction in total CD8^+^ T cell cytotoxic effector molecules is driven by the reduction in the frequencies of EM CD8^+^ T cells under these conditions. Conversely, when memory recall responses are specifically induced through stimulation with MHC-I restricted antigens, EM and CM CD8^+^ T cells in JSLE appear to be hypersensitive to stimulation. Interestingly, it has been reported that human naïve CD8^+^ T cells primed in the presence of IFN-α exhibit a heightened ability to respond to secondary antigen stimulation [79]. As excessive production of IFN-α has been shown to play a central role in the pathogenesis of SLE [80], it is possible that increased exposure to IFN-α could be driving the apparent heightened CD8^+^ T cell antigenic memory recall responses to viral peptides seen in JSLE. This also highlights the fact that reductions in cytotoxic populations do not necessarily imply that cytotoxic cell responses are diminished in JSLE, and further investigations are necessary using various experimental conditions to confirm the functional status of peripheral blood CD8^+^ T cell populations in JSLE.

The possibility that there may be a defect in CD8^+^ T cell effector function in JSLE raises the question of why this could be the case. Transcriptomic analysis of CD8^+^ T cells from JSLE and HC, aimed at providing some potential answers to this question, identified upregulation of IFN-α responses and possible metabolic and mitochondrial disturbances in CD8^+^ T cells in JSLE. Numerous transcriptomic studies in adult-onset SLE have identified a type I IFN gene signature (IGS) in multiple cell populations, with some groups reporting an association between type 1 IGS and disease activity, while others were unable to validate this association (reviewed in [81]). Although transcriptomic studies in JSLE are very limited, the above mentioned transcriptomic study describing single-cell RNA sequencing in 33 paediatric lupus patients showed that a high IFN signature was limited to a small number of cells within each major cell population and subpopulations with high IGS were expanded in active JSLE [20]. Our findings that IFN-α stimulated genes are upregulated in CD8^+^ T cells in JSLE are in keeping with these published observations, though in our analysis this was associated with JSLE regardless of disease activity.

Abnormal mitochondrial physiology (reviewed in [82]) has been well described in multiple studies in SLE. In recent years, much attention has focused on investigating the bioenergetic profile of CD8^+^ T cells and it is now widely recognized that the nutrient environment that cells are exposed to may affect their developmental trajectory and determine their ability to function as effectors [83]. Our analysis of non-lipid serum metabolite levels in JSLE patients and HC revealed a reduction in ketone bodies, pyruvate, and glycerol in JSLE which could point to metabolic disturbances and reduction in glycolysis, oxidative phosphorylation, and ATP energy production in tissues in JSLE. Positive correlations between frequencies of EM CD8^+^ T cells and levels of ketone bodies in JSLE and mitochondrial scores in HC, suggest there may be a link between EM CD8^+^ T cell mitochondrial function and reduction in CD8^+^ T cell cytotoxicity in JSLE. However, the changes in serum metabolite levels are more likely to be a reflection of differences in global metabolism in tissues, such as muscle, and less likely to reflect lymphocyte metabolism. In addition, medications commonly used to treat SLE, steroids in particular, can influence the same metabolic pathways [84]. Nevertheless, metabolic abnormalities have been reported in CD8^+^ T cells in adult-onset SLE and have been attributed both to prolonged type I IFN exposure and T cell receptor (TCR) stimulation and also to mitophagy inhibition through CD38 expression leading to reduction in CD8^+^ T cell cytotoxicity and altering the function and morphology of mitochondria in adult-onset SLE [23, 32, 33]. Modulating CD8^+^ T cell metabolic function has also been postulated as a method of controlling over-active effector response to chronic viral infection which when unchecked could lead to autoimmunity [85]. In accordance with the data in adult-onset SLE which linked increased mass and size of mitochondria with impaired mitophagy [86], our experiments assessing CD8^+^ T cell mitochondrial morphology showed increased mitochondrial volume and surface area in JSLE. Expression of mitochondrial-encoded genes was reduced and mitochondrial score correlated negatively with type I IGS in JSLE. Mitochondrial dysfunction has been shown to induce IGS expression in monocytes from patients with juvenile dermatomyositis [42] and it is possible that the same mechanism could be driving EM cell apoptosis resulting in reduced CD8^+^ T cell cytotoxic capacity in JSLE. Additionally, it has recently been demonstrated that sustained killing by CD8^+^ T cells requires mitochondrial translation, as inhibiting mitochondrial translation resulted in CD8^+^ T cells that were unable to fill lytic granules with cytoplasmic cytotoxic proteins [87]. This possible direct link between mitochondrial abnormalities and reduced cytotoxic capacity of CD8^+^ T cells in JSLE would be an interesting subject for future research.

Notable limitations of this study include the moderate JSLE cohort sample size due to the rarity of the condition and restricted opportunities to obtain numerous samples from children and adolescents. Another significant limitation is that the patients were not treatment-naïve, preventing us from gaining a full understanding of the severity of cytotoxic CD8^+^ T cell abnormalities at disease onset. Additionally, conducting detailed functional experiments is inherently difficult in JSLE due to lymphopenia, particularly in active disease. Full blood counts were unavailable for our healthy control cohort which meant that it was not possible to compare absolute cell numbers of cytotoxic populations. Assessment of cytolytic CD8^+^ T cell function was hampered by the absence of reliable assay techniques and lack of identified CD8^+^ T cell TCR autoantigen specificities in JSLE, while our reliance on frozen, bio-banked samples prevented detailed assessment of mitochondrial function. Furthermore, our findings are limited to the CD8^+^ T cell phenotype and function in peripheral blood. Analysis of tissues from organs affected by the disease is required to determine if cytotoxic CD8^+^ T cells contribute to disease pathology at those disease sites.

To conclude, we have shown that cytotoxic CD8^+^ T cell populations are reduced in this representative cohort of JSLE patients. This reduction, as well as reduction of cytotoxic mediators and cytokines in serum of JSLE patients, represents a deviation from the normal immune phenotype and further work is necessary to determine the functional implications of this in JSLE. Aberrant cytotoxic function may be implicated in the disease pathogenesis at onset or may be a consequence of disease progression, as well as reflect the impact of infections or treatment in JSLE which is notoriously heterogeneous. Our studies also add to the growing evidence that mitochondrial defects are linked to enhanced type I IFN signalling and are a common underlying pathogenic mechanism impacting a wide range of altered immune phenotypes in adult-onset SLE and now JSLE. Considering the growing range of type I IFN targeting therapies in clinical trials for SLE treatment, and the interest in developing mitochondrial-targeting therapies, our data potentiates that these agents would be similarly effective in juvenile disease which is often neglected in the development of new therapies compared to its adult counterpart.

## Supplementary Material

uxae127_suppl_Supplementary_Materials

## Data Availability

RNA sequencing data can be found at ArrayExpress: EMBL-EBI (www.ebi.ac.uk/arrayexpress), accession number: E-MTAB-14531. This data will be available from manuscript publication date.
