# Supplementary material for: Type I interferon and mitochondrial dysfunction are associated with dysregulated cytotoxic CD8+ T cell responses in juvenile systemic lupus erythematosus
Source: Clin Exp Immunol. 2024 Dec 25;219(1):uxae127. doi: 10.1093/cei/uxae127 (PMC11748002; doi:10.1093/cei/uxae127)
Supplement: uxae127_suppl_Supplementary_Materials [file uxae127_suppl_Supplementary_Materials.docx]

Supplementary Material

**Type I interferon and mitochondrial dysfunction are associated with dysregulated cytotoxic CD8^+^ T cell responses in juvenile systemic lupus erythematosus**

[Supplementary Figure S1. CD8^+^ T cell degranulation capacity may be diminished in JSLE. 3](#_Toc178616030)

[Supplementary Figure S2. Reduction in CD8^+^ T cell cytotoxicity does not correlate with JSLE disease activity. 4](#_Toc178616031)

[Supplementary Figure S3. Reductions in CD8^+^ T cell cytotoxic and cytokine producing populations are not associated with JSLE BILAG clinical disease activity. 6](#_Toc178616032)

[Supplementary Figure S4. Reductions in CD8^+^ T cell populations are not associated with drug treatment. 7](#_Toc178616033)

[Supplementary Figure S5. Correlations between cytotoxic and cytokine producing CD8^+^ T cells and haematological and serological markers of disease activity in JSLE. 9](#_Toc178616034)

[Supplementary Figure S6. CD8^+^ T cell cytotoxic capacity does not change with age. 11](#_Toc178616035)

[Supplementary Figure S7. No sex differences in frequencies of perforin^+^, IFN-γ^+^, and TNF-α^+^ CD8^+^ T cells in JSLE and HC. 12](#_Toc178616036)

[Supplementary Table S1. Demographic characteristics of healthy subjects and JSLE patients in CD8^+^ T cell transcriptional analysis. 13](#_Toc178616037)

[Supplementary Table S2. Clinical characteristics of JSLE patients included in transcriptional analysis cohort. 14](#_Toc178616038)

[Supplementary Table S3. Comparison of demographic and disease activity characteristics between CD8^+^ T cell immunophenotyping and transcriptional analysis cohorts. 15](#_Toc178616039)

[Supplementary Table S4. Top 10 upregulated and downregulated CD8^+^ T cell genes in JSLE patients compared to healthy controls. 16](#_Toc178616040)

[Supplementary Figure S8. GSEA of CD8^+^ T cell transcriptomic data confirms upregulation of IFN-α responses and potential changes in mitochondrial function in JSLE. 17](#_Toc178616041)

[Supplementary Figure S9. No differences in total CD8^+^ T cell death in JSLE vs HC with and without IFN-α stimulation. 18](#_Toc178616042)

[Supplementary Figure S10. No differences in perforin or cytokine expression in CD8^+^ T cell subsets in JSLE vs HC. 20](#_Toc178616043)

[Supplementary Figure S11. Positive correlations between frequencies of EM CD8^+^ T cells and ketone body measurements in JSLE and mitochondrial score in HC. 21](#_Toc178616044)

**
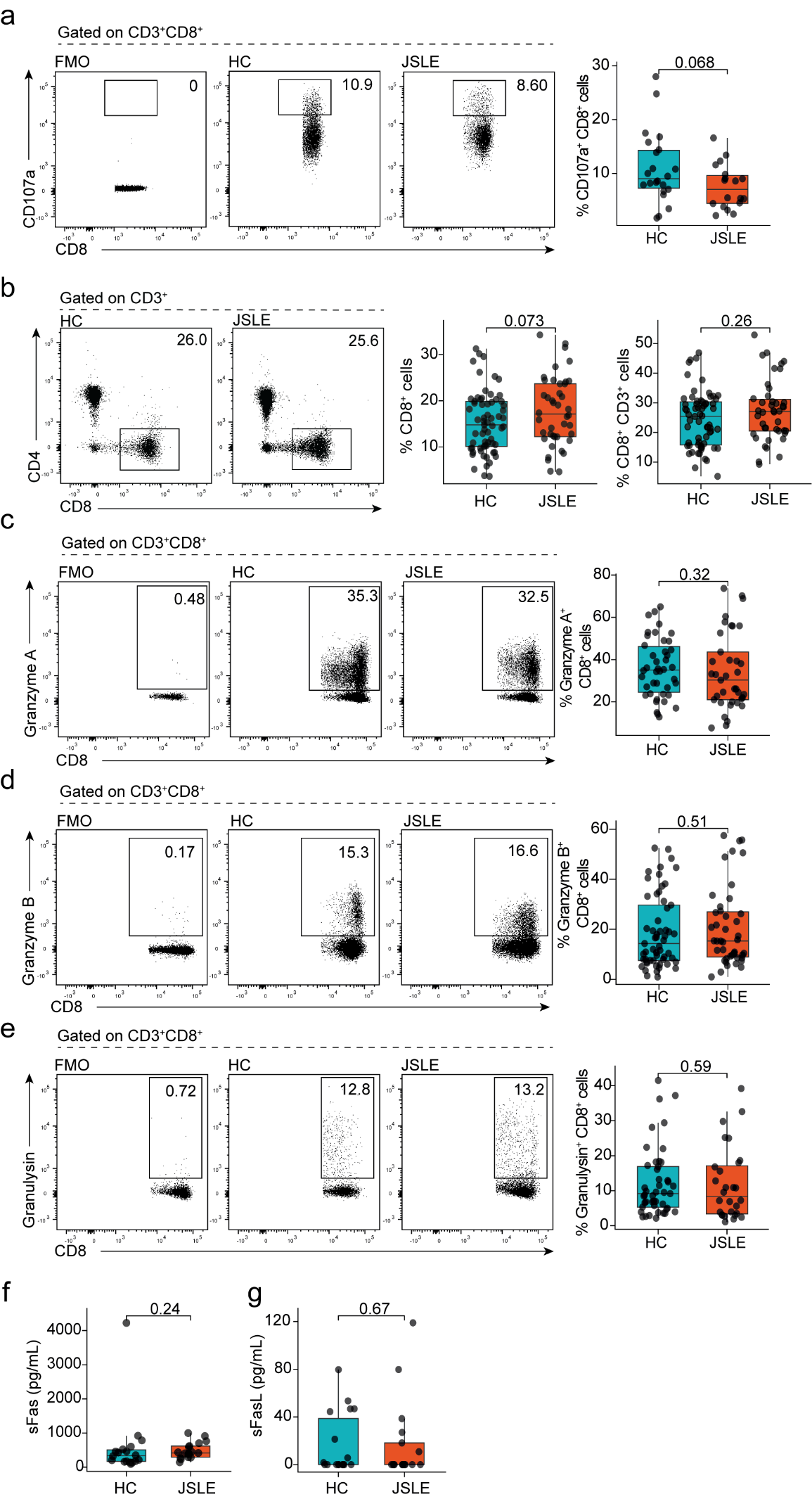
**

Supplementary Figure S1. CD8^+^ T cell degranulation capacity may be diminished in JSLE.

**(a)** Representative flow plots and box plot showing percentage of CD8^+^ T cells expressing CD107a in PBMCs from HC (n=22) and JSLE (n=18) cultured with PMA/ionomycin in the presence of brefeldin and monensin for 4 hours. **(b)** Representative flow diagrams and box plots showing the frequency of CD8^+^ T cells in HC (n=65) and JSLE (n=42) expressed as a percentage of live cells (left) and as a percentage of CD3^+^ cells (right). Representative flow plots and box plots showing percentage of CD8^+^ T cells in *ex-vivo* PBMCs expressing **(c)** granzyme A (HC n=42, JSLE n=37), **(d)** granzyme B (HC n=57, JSLE n=43), and **(e)** granulysin (HC n=45, JSLE n=28) in HC and JSLE. Numbers inside gates in flow plots indicate percentage of cells. Boxplots quantifying levels of **(f)** sFas, and **(g)** sFasL in serum of HC (n=18) and JSLE patients (n=17). Analytes were measured using a flow cytometry-based bead multiplex assay. All boxplots show median ± IQR. p-values calculated using unpaired Mann-Whitney U test **(b-f)** or unpaired t-test as appropriate **(a)**. HC=healthy controls, IQR=interquartile range, JSLE=juvenile systemic lupus erythematosus, PBMCs=peripheral blood mononuclear cells, sFas=soluble Fas, sFasL=soluble Fas ligand.


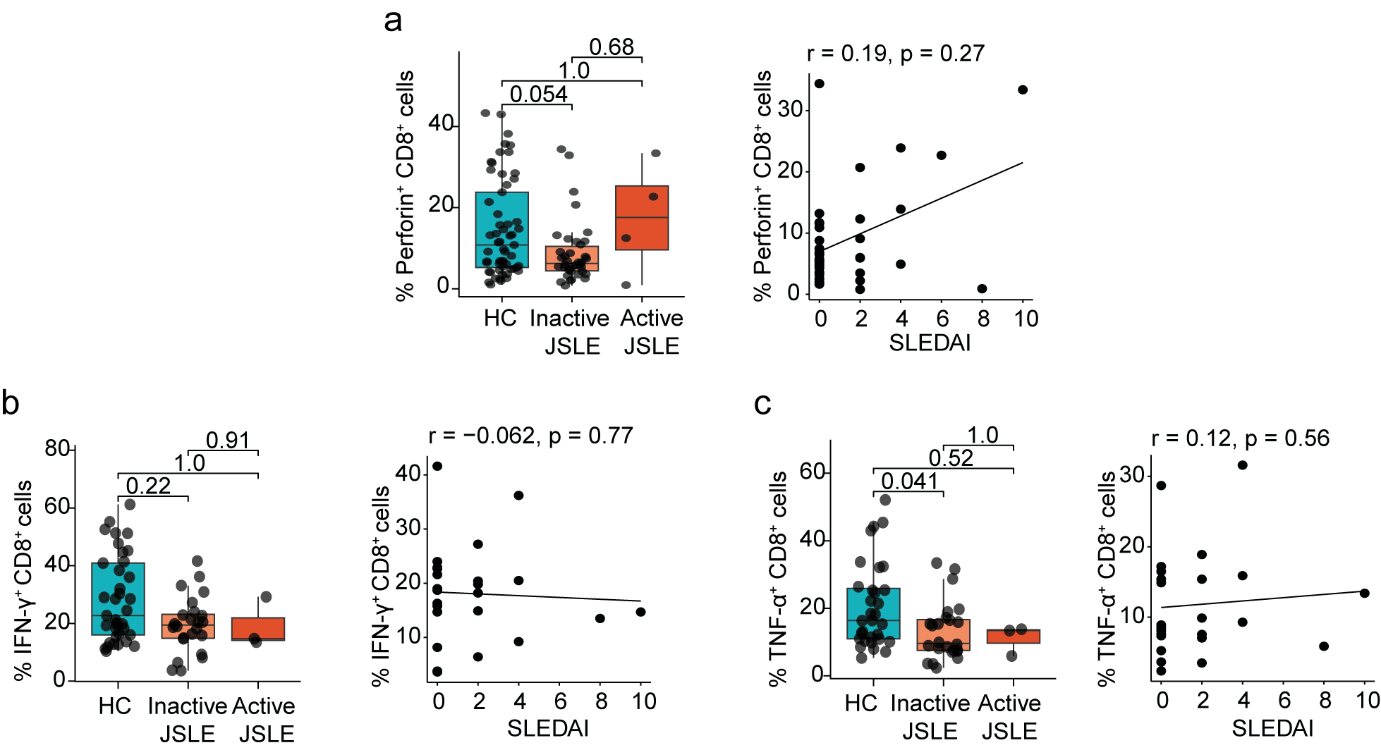


Supplementary Figure S2. Reduction in CD8^+^ T cell cytotoxicity does not correlate with JSLE disease activity.

Boxplots of frequencies of **(a)** perforin^+^ CD8^+^ T cells (HC n=57, Inactive JSLE n=38, Active JSLE n=4), **(b)** IFN-γ^+^ CD8^+^ T cells (HC n=37, Inactive JSLE n=24, Active JSLE n=3), and **(c)** TNF-α^+^ CD8^+^ T cells (HC n=31, Inactive JSLE n=24, Active JSLE n=3) stratified based on SLEDAI score. Active JSLE=SLEDAI >4, Inactive JSLE=SLEDAI <= 4. Boxplots show median ± IQR. p-values calculated using Dunn’s test with Bonferroni correction. Scatter plots showing correlations between JSLE SLEDAI scores and frequencies of **(a)** perforin^+^ (n=36), **(b)** IFN-γ^+^ (n=24), **(c)** TNF-α^+^ (n=24) CD8^+^ T cells. Spearman’s rho correlation coefficients and their associated p-values are shown. HC=healthy controls, IFN=interferon, IQR=interquartile range, JSLE=juvenile systemic lupus erythematosus, SLEDAI=Systemic Lupus Erythematosus Disease Activity Index-2K, TNF=tumour necrosis factor.

**
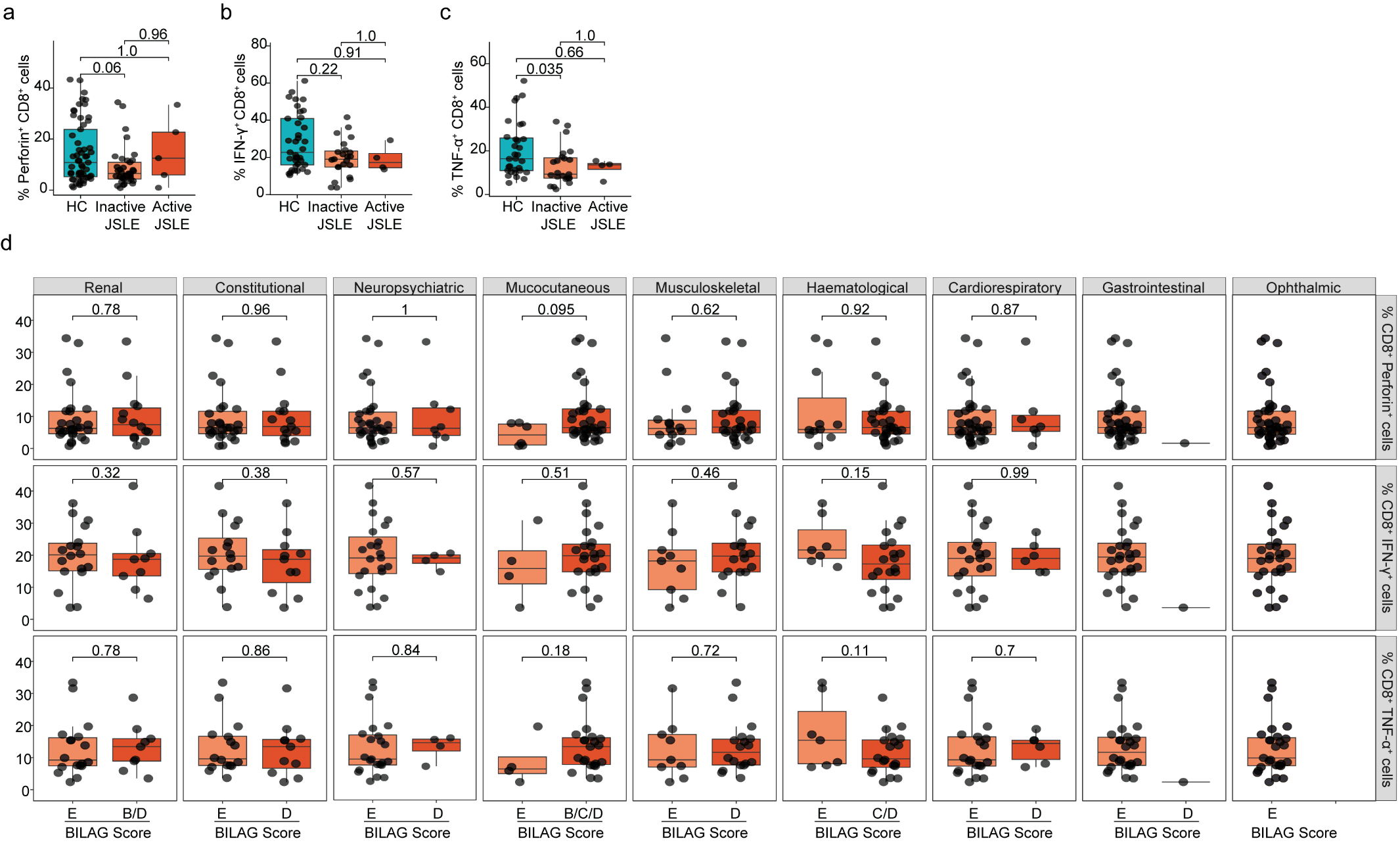
**

Supplementary Figure S3. Reductions in CD8^+^ T cell cytotoxic and cytokine producing populations are not associated with JSLE BILAG clinical disease activity.

Boxplots showing frequencies of **(a)** perforin^+^ (HC n=57, Inactive JSLE n=37, Active JSLE n=5), **(b)** IFN-γ^+^ (HC n=37, Inactive JSLE n=23, Active JSLE n=4), and **(c)**TNF-α^+^ (HC n=31, Inactive JSLE n=23, Active JSLE n=4) CD8^+^ T cells stratified based on global BILAG score. Active disease was defined as at least one BILAG B score (Global BILAG score ≥8) in any of the organ domains. P-values calculated using Dunn’s multiple comparison test with Bonferroni correction. **(d)** Boxplots showing frequencies of perforin^+^, IFN-γ^+^ and TNF-α^+^ CD8^+^ T cells in JSLE patients stratified into those with current or previous organ involvement (BILAG scores A-D) and those who never experienced disease involvement in the specified organ domain (BILAG score E) across the 9 organ systems comprising the BILAG score. All boxplots show median ± IQR. p values calculated using unpaired two-sided t-test (IFN-γ: all domains, TNF-α: haematological) or unpaired Mann-Whitney U test (perforin: all domains, TNF-α: renal, constitutional, neuropsychiatric, mucocutaneous, musculoskeletal, cardiorespiratory), as appropriate based on distribution of the data. BILAG= British Isles Lupus Activity Group, HC=healthy controls, IFN=interferon, IQR=interquartile range, JSLE=juvenile systemic lupus erythematosus, TNF=tumour necrosis factor.


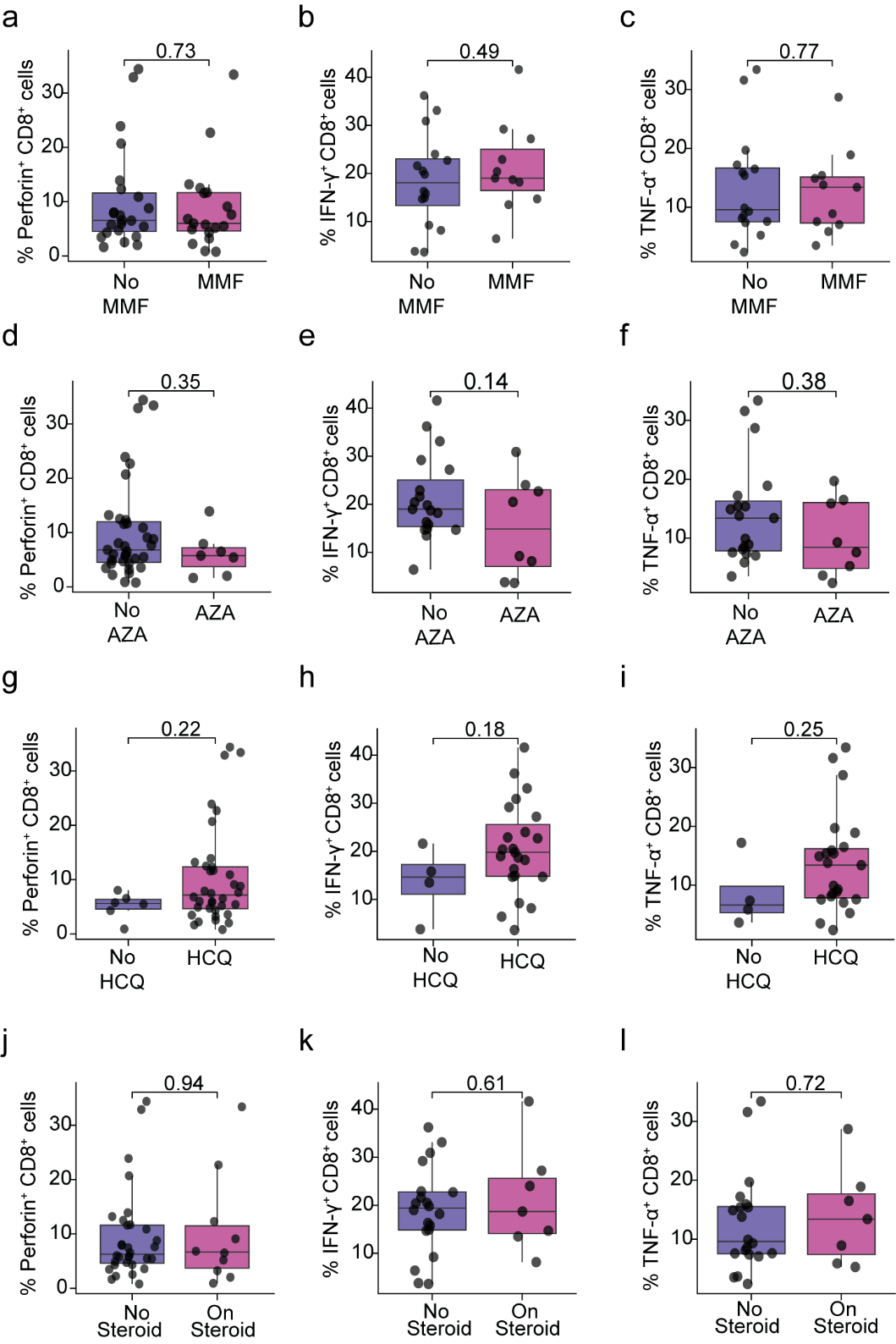


Supplementary Figure S4. Reductions in CD8^+^ T cell populations are not associated with drug treatment.

Boxplots showing frequencies of perforin, IFN-γ, and TNF-α expressing CD8^+^ T cells stratified on treatment with **(a, b, c)** MMF (CD8^+^perforin^+^: no MMF n=23, MMF n=19, CD8^+^IFN-γ^+^/CD8^+^TNF-α^+^: no MMF n=16, MMF n=11), **(d, e, f)** AZA (CD8^+^perforin^+^: no AZA n=35, AZA n=7, CD8^+^IFN-γ^+^/CD8^+^TNF-α^+^: no AZA n=19, AZA n=8), **(g, h, i)** HCQ (CD8^+^perforin^+^: no HCQ n=6, HCQ n=36, CD8^+^IFN-γ^+^/CD8^+^TNF-α^+^: no HCQ n=4, HCQ n=23) and **(j, k ,l)** oral prednisolone use at time of sampling (CD8^+^perforin^+^: no steroid n=32, steroid n=10, CD8^+^IFN-γ^+^/CD8^+^TNF-α^+^: no steroid n=20, steroid n=7). All boxplots show median ± IQR. p-values were calculated using unpaired two-sided Student’s t-test **(b, e, h, k)** or Mann-Whitney U test **(a, c, d, f, g, i, j, l)** as appropriate based on the data distribution. AZA=azathioprine, HCQ=hydroxychloroquine, IFN=interferon, IQR=interquartile range, MMF=mycophenolate mofetil, TNF=tumour necrosis factor.


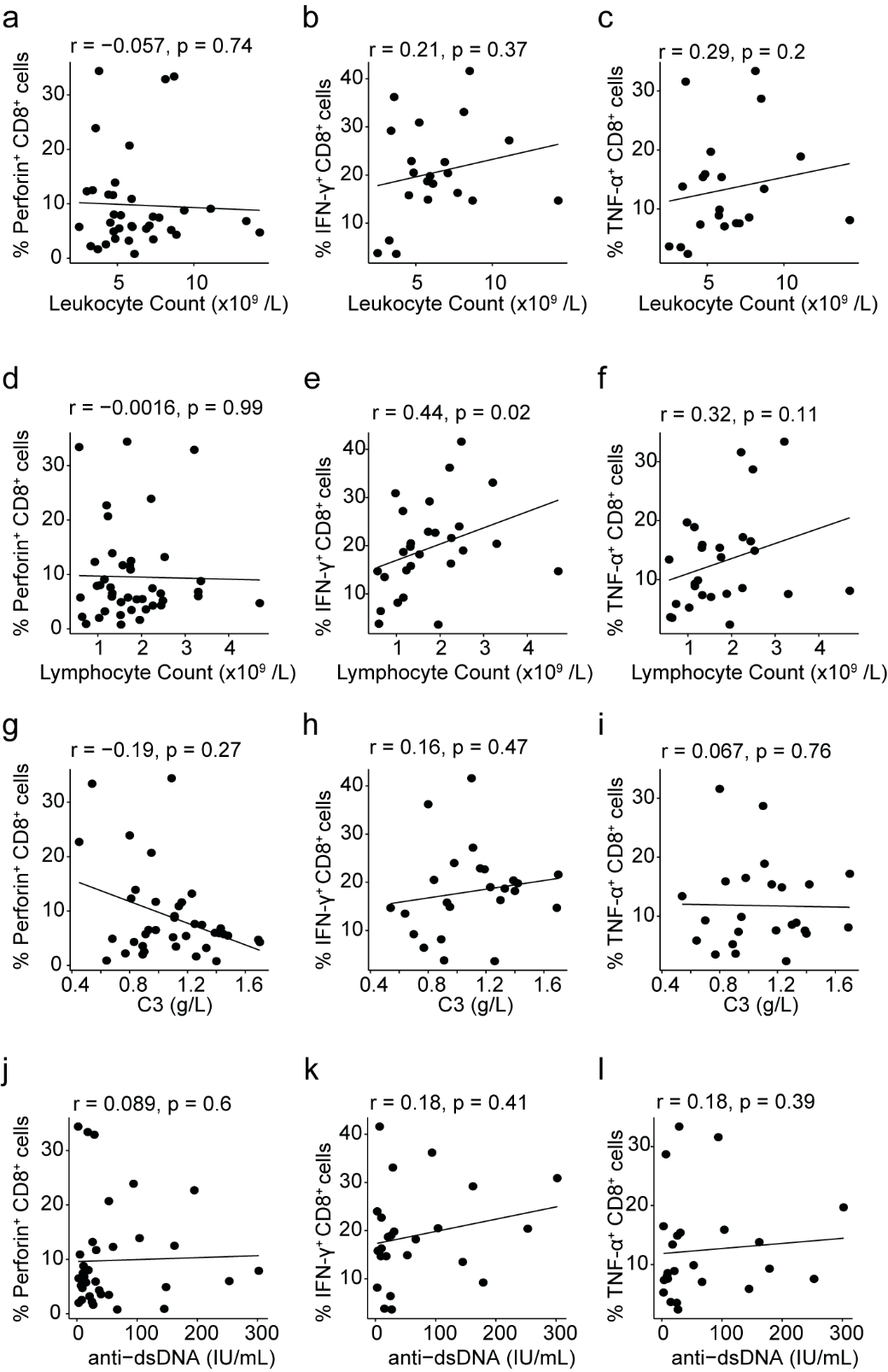


Supplementary Figure S5. Correlations between cytotoxic and cytokine producing CD8^+^ T cells and haematological and serological markers of disease activity in JSLE.

Scatter plots showing correlations in JSLE between leukocyte counts and **(a)** perforin^+^ (n=36), **(b)** IFN-γ^+^(n=21) and **(c)** TNF-α^+^ (n=21) CD8^+^ T cell frequencies. Scatter plots showing correlations in JSLE between lymphocyte count and **(d)** perforin^+^ (n=42), **(e)** IFN-γ^+^ (n=27) and **(f)** TNF-α^+^ (n=27) CD8^+^ T cell frequencies. Scatter plots showing correlations between levels of C3 and **(g)** perforin^+^ (n=38), **(h)** IFN-γ^+^(n=24) and **(i)** TNF-α^+^ (n=24) CD8^+^ T cell frequencies and between anti-dsDNA titres and **(j)** perforin^+^ (n=37), **(k)** IFN-γ^+^ (n=24) and **(l)** TNF-α^+^ (n=24) CD8^+^ T cell frequencies in JSLE patients. Spearman’s rho **(a, c-g, i-l)** or Pearson r **(b, h)** correlation coefficients and their associated p-values are shown. anti-dsDNA=anti-double stranded DNA antibodies, C3=complement 3, IFN=interferon, TNF=tumour necrosis factor.


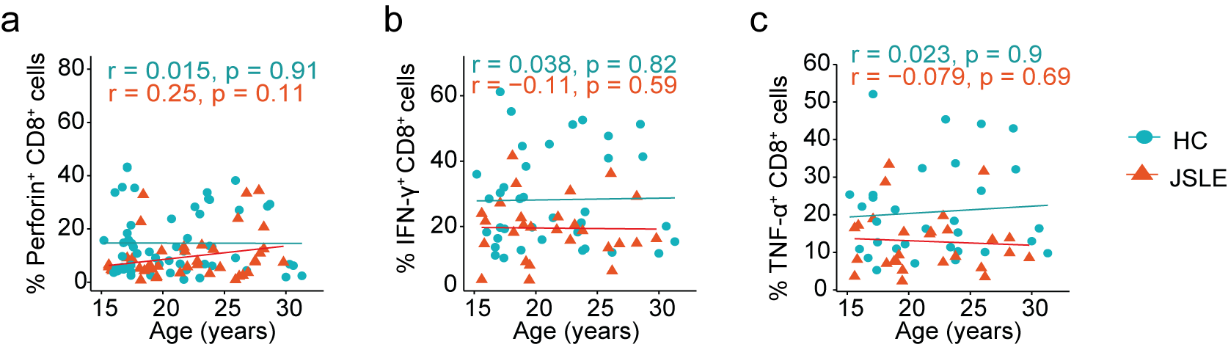


Supplementary Figure S6. CD8^+^ T cell cytotoxic capacity does not change with age.

Scatter plots showing correlations between age at time of sampling and frequencies of **(a)** perforin (HC n=57, JSLE n =42), **(b)** IFN-γ (HC n=37, JSLE n=27) or **(c)** TNF-α (HC n=31, JSLE n=27) expressing CD8^+^ T cells. Spearman’s rho correlation coefficients and the associated p-values are shown. HC=healthy controls, IFN=interferon, JSLE=juvenile systemic lupus erythematosus, TNF=tumour necrosis factor.


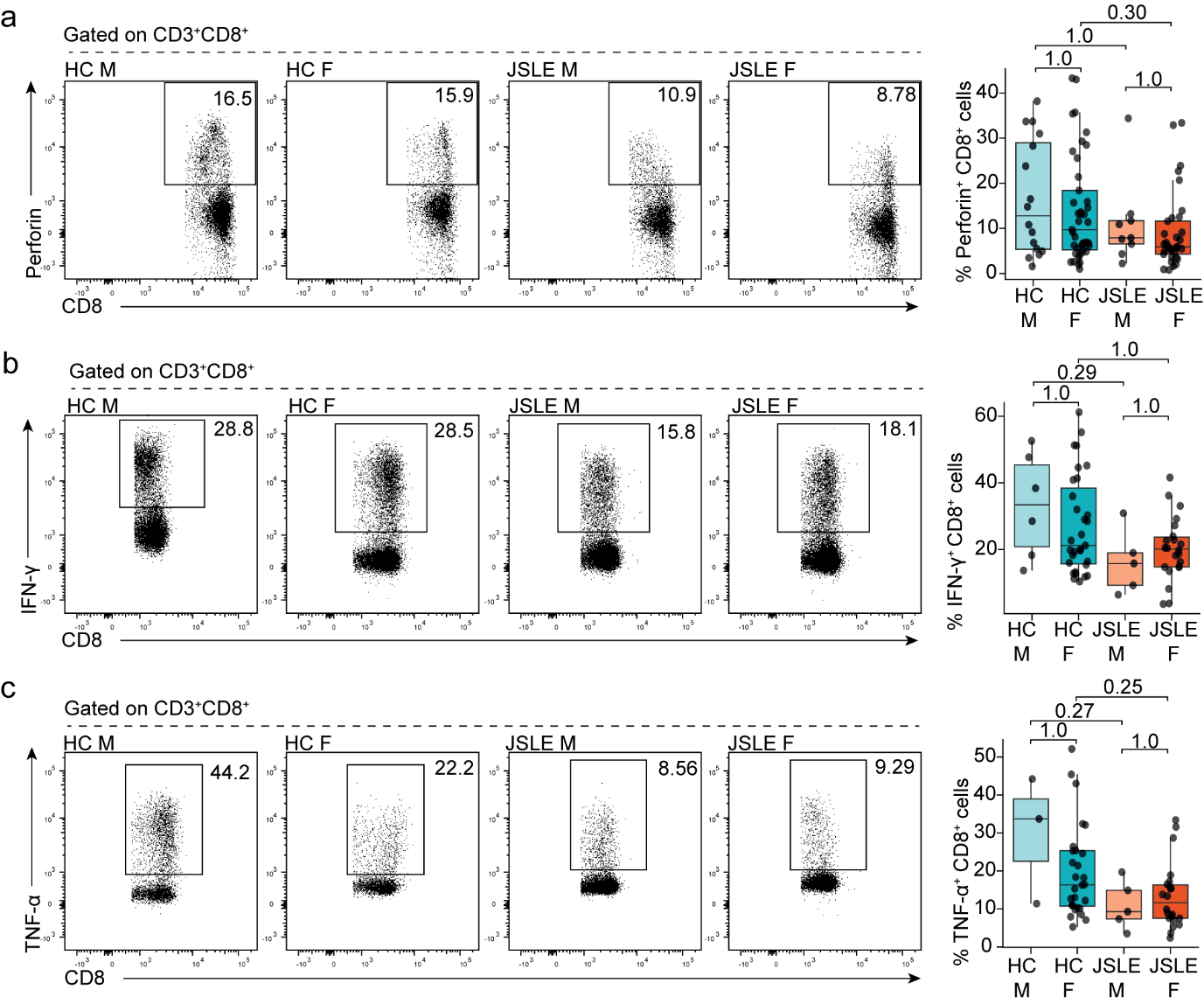


Supplementary Figure S7. No sex differences in frequencies of perforin^+^, IFN-γ^+^, and TNF-α^+^ CD8^+^ T cells in JSLE and HC.

Representative flow diagrams and boxplots quantifying **(a)** perforin^+^ (HC M: n=16, HC F: n=41, JSLE M: n=9, JSLE F: n=33), **(b)** IFN-γ^+^ (HC M: n=6, HC F: n=31, JSLE M: n=5, JSLE F: n=22), **(c)** TNF-α^+^ (HC M: n=3, HC F: n=28, JSLE M: n=5, JSLE F: n=22) frequencies of CD8^+^ T cells across self-reported sex. Numbers in flow plot gates indicate percentage of cells. Boxplots show median ± IQR. p-values calculated using Dunn’s test with Bonferroni correction. F=female, HC=healthy controls, IFN=interferon, IQR=interquartile range, JSLE=juvenile systemic lupus erythematosus, M=male, TNF=tumour necrosis factor.

Supplementary Table S1. Demographic characteristics of healthy subjects and JSLE patients in CD8^+^ T cell transcriptional analysis.

|  | **HC**  Number  (% / range) | **JSLE**  Number  (% / range) | **p-value** |
| --- | --- | --- | --- |
| Total number | 29 | 26 | - |
| Female:Male | 18:11 | 14:12 | 0.59 |
| Median age (years) | 20.9 (15.1-25.4) | 20.5 (15.4-28.2) | 0.46 |
| **Ethnicity** |  |  |  |
| White | 16 (55%) | 9 (35%) | 0.18 |
| South Asian | 6 (21%) | 8 (31%) | 0.54 |
| East Asian | 4 (14%) | 1 (4%) | 0.36 |
| Black | 1 (3%) | 4 (15%) | 0.18 |
| Other | 2 (7%) | 4 (15%) | 0.41 |

P-values calculated using Fisher’s exact test (sex and ethnicity) or Mann Whitney test (age). HC=healthy controls, JSLE=juvenile systemic lupus erythematosus.

Supplementary Table S2. Clinical characteristics of JSLE patients included in transcriptional analysis cohort.

| **Clinical Features^*†^** | **Number (% / range)** |
| --- | --- |
| Median disease duration (years) | 7.9 |
| Median age at onset (years) | 12.3 |
| Average SLEDAI, n=22 | 2.5 (0-10) |
| SLEDAI = 0-4 | 21 (81%) |
| SLEDAI = 6-10 | 5 (19%) |
| Average BILAG | 3 (0-24) |
| Global BILAG = 0 | 17 (65%) |
| Global BILAG =1 (1 score C) | 3 (12%) |
| Global BILAG = 8 (1 score B) | 3 (12%) |
| Global BILAG = 9 (1 score B + 1 score C) | 1 (4%) |
| Global BILAG = 18 (2 score B) | 1 (4%) |
| Global BILAG = 24 (3 score B) | 1 (4%) |
| Renal Involvement | 7 (27%) |
| Constitutional Involvement | 8 (31%) |
| Neuropsychiatric Involvement | 4 (15%) |
| Mucocutaneous Involvement | 22 (85%) |
| Musculoskeletal Involvement | 18 (69%) |
| Haematological Involvement | 4 (15%) |
| Cardiorespiratory Involvement | 4 (15%) |
| Gastrointestinal Involvement | 0 (0%) |
| Ophthalmic Involvement | 0 (0%) |
| **Serology^*^** | **Median** |
| anti-dsDNA (IU/mL) (NR=<50), n=22 | 34.5 |
| C3 (g/L) (NR=0.9-1.8), n=24 | 1.0 |
| Lymphocyte count (10^9^ /L) (NR=1.2-3.5) | 1.6 |
| Leukocyte count (10^9^ /L) (NR=3-10), n=16 | 6.4 |
| **Treatment^*^** | **Number (%)** |
| None | 1 (4%) |
| Rituximab in the past year | 0 (0%) |
| Rituximab ever | 4 (15%) |
| Average duration since last rituximab treatment (years) | 5.9 |
| Hydroxychloroquine | 24 (92%) |
| Methotrexate | 2 (8%) |
| Azathioprine | 3 (12%) |
| Mycophenolate Mofetil | 13 (50%) |
| Prednisolone (any dose) | 5 (19%) |
| Prednisolone >=10mg/day | 3 (12%) |
| Cyclophosphamide in the past year | 1 (4%) |

*Data is presented for the entire cohort (n=26), unless stated otherwise. ^†^Organ involvement includes patients with current or previous disease activity in the specified organ domain. anti-dsDNA=anti- double stranded DNA antibody, BILAG=British Isles Lupus Activity Group global score, C3=complement 3, SLEDAI=Systemic lupus erythematosus disease activity index-2K, NR=normal range.

Supplementary Table S3. Comparison of demographic and disease activity characteristics between CD8^+^ T cell immunophenotyping and transcriptional analysis cohorts.

|  | **Phenotyping Cohort** | **RNA-seq Cohort** | **p-value** |
| --- | --- | --- | --- |
| **Number HC/JSLE** | 68/44 | 29/26 | - |
| **Sex ratio F:M** |  |  |  |
| HC | 46:22 | 18:11 | 0.64 |
| JSLE | 34:10 | 14:12 | 0.06 |
| **Median age in years (range)** |  |  |  |
| HC | 20.1 (15.2-32.2) | 20.9 (15.1-25.4) | 0.90 |
| JSLE | 21.6 (15.6-29.8) | 20.5 (15.4-28.2) | 0.14 |
| **Disease activity measures** |  |  |  |
| Average SLEDAI (range) | 1.6 (0-10) | 2.5 (0-10) | 0.20 |
| Average BILAG (range) | 1.0 (0-9) | 3 (0-24) | 0.16 |

P-values calculated using Fisher’s exact test (sex) and unpaired Mann-Whitney U test (age, SLEDAI, BILAG). BILAG=British Isles Lupus Activity Group global score, HC=healthy controls, JSLE=juvenile systemic lupus erythematosus, RNA-seq=ribonucleic acid sequencing, SLEDAI=Systemic lupus erythematosus disease activity index-2K

Supplementary Table S4. Top 10 upregulated and downregulated CD8^+^ T cell genes in JSLE patients compared to healthy controls.

| **Gene Name** | **Gene Function** | **Adjusted p-value** | **Log2**  **Fold Change** |
| --- | --- | --- | --- |
| Upregulated (FDR adjusted p-value < 0.05, FC>0) | | | |
| IFI27 | Interferon-alpha induced, pro-apoptotic, mitochondrial membrane protein | 1.18E-18 | 4.67 |
| LAMP3 | Trafficking of proteins to lysosomes, modulation of autophagy | 1.55E-16 | 2.44 |
| IFI44L | Interferon-induced antiviral response | 1.56E-15 | 3.90 |
| IFI6 | Interferon-induced antiviral response, negative regulation of intrinsic apoptosis | 1.26E-13 | 1.96 |
| USP18 | Ubiquitin-specific protease, downregulation of interferon response | 1.33E-12 | 2.16 |
| RSAD2 | Interferon-induced antiviral response | 1.33E-12 | 2.32 |
| OTOF | Vesicle membrane fusion | 2.51E-12 | 3.59 |
| OAS3 | Interferon-induced antiviral response | 1.49E-10 | 1.76 |
| IFI44 | Interferon-induced antiviral response | 1.73E-10 | 2.42 |
| ISG15 | Interferon-induced antiviral response | 2.33E-10 | 1.72 |
| Downregulated (FDR adjusted p-value < 0.05, FC<0) | | | |
| PLCB1 | Phospholipase (phospholipid hydrolysis) | 1.90E-06 | -1.02 |
| AC022239.1 | lncRNA, function unknown | 2.15E-06 | -1.93 |
| WNT11 | Developmental processes, regulation of cell fate and patterning during embryogenesis (probable) | 0.00014 | -1.78 |
| ELOVL4 | Fatty acid biosynthesis | 0.00015 | -1.01 |
| AC253572.1 | lncRNA, function unknown | 0.00036 | -1.42 |
| AC245014.3 | lncRNA, function unknown | 0.00039 | -1.08 |
| AC109326.1 | lincRNA, function unknown | 0.00054 | -1.21 |
| KLRB1 | T cell co-stimulation and co-inhibition (CD161) | 0.00095 | -1.32 |
| RNVU1-27 | snRNA, function unknown | 0.00095 | -0.92 |
| HADHA | Mitochondrial beta-oxidation of long chain fatty acids | 0.0014 | -0.26 |

FC=fold change, FDR=false discovery rate.


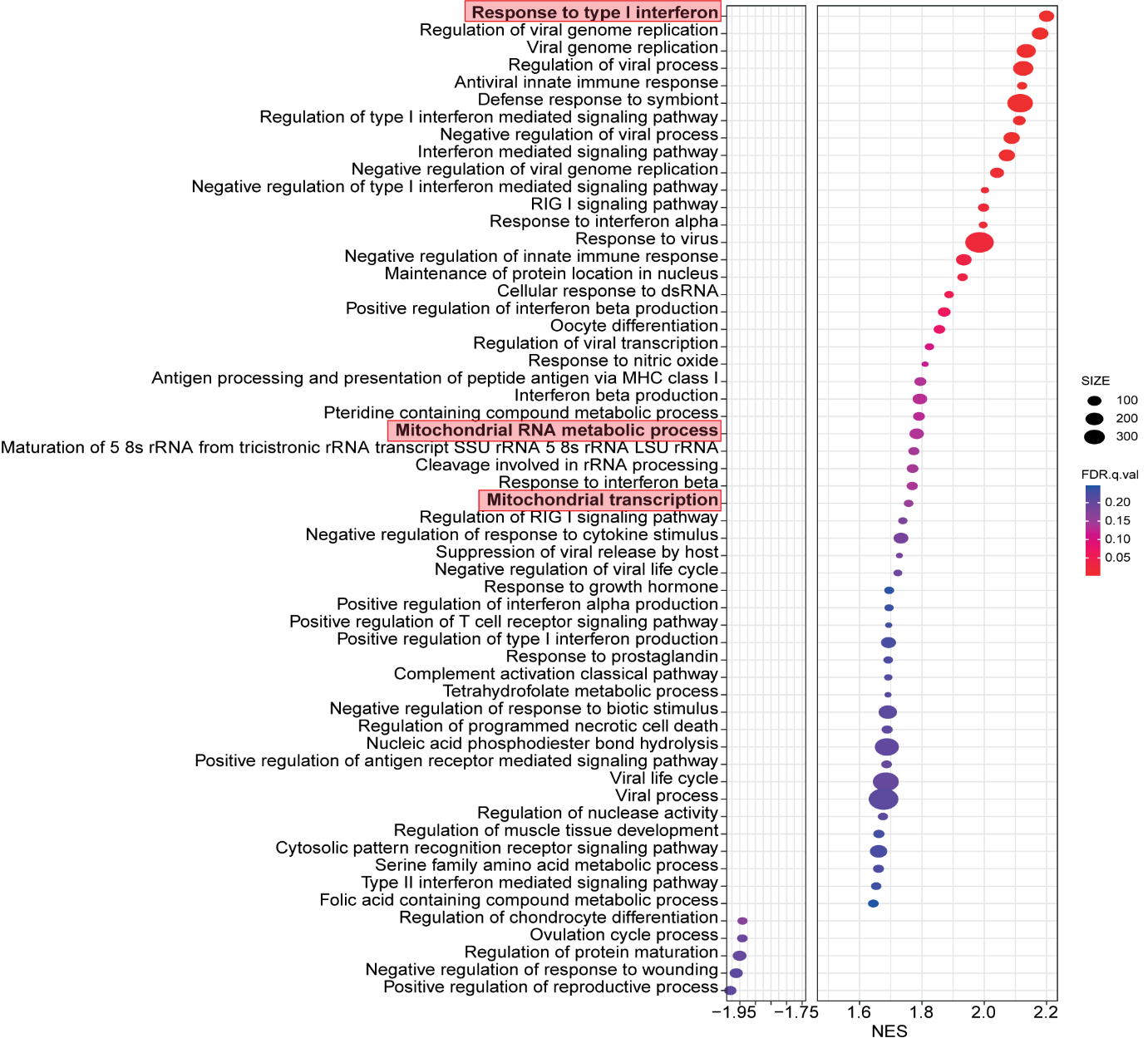


Supplementary Figure S8. GSEA of CD8^+^ T cell transcriptomic data confirms upregulation of IFN-α responses and potential changes in mitochondrial function in JSLE.

Dot plot of significantly enriched terms identified in GSEA performed using phenotype permutation and the GO BP ontology catalogue. Plot shows FDR q.value (p-value normalised for gene set size and multiple testing) and normalized enrichment scores (NES) of pathway ontology terms in CD8^+^ T cells of JSLE vs HC using the entire gene list ranked by signal-to-noise ratio. Positive NES score indicates enrichment in JSLE compared to HC, negative NES score indicates enrichment in HC vs JSLE. Dot size indicates number of genes in the gene set. Only pathways meeting threshold for significance (FDR q.value < 0.25) are shown. dsRNA=double stranded ribonucleic acid, FDR=false discovery rate, GO BP=gene ontology biological process, GSEA=gene set enrichment analysis, HC=healthy controls, IFN=interferon, JSLE=juvenile systemic lupus erythematosus, MHC=major histocompatibility complex, NES=normalised enrichment scores, RIG-I=Retinoic Acid Inducible Gene I, rRNA=ribosomal ribonucleic acid.


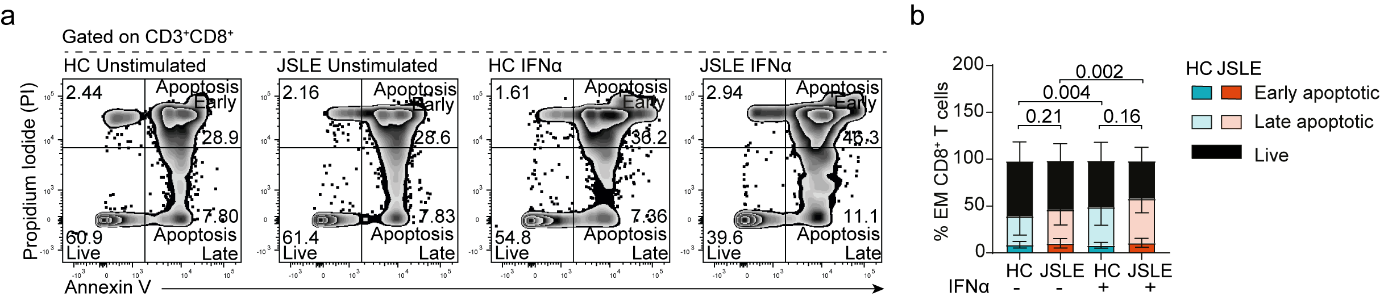


Supplementary Figure S9. No differences in total CD8^+^ T cell death in JSLE vs HC with and without IFN-α stimulation.

Representative flow plots **(a)** showing Annexin V and PI staining. Numbers in quadrants indicate percentages of cells. **(b)** Stacked bar plot quantifying frequencies of early apoptotic, late apoptotic and live total CD8^+^ T cells in HC (n=9) and JSLE patients (n=10) in PBMCs with and without stimulation with IFN-α for 48 hours. Bar plot displays means ± SD. p-values comparing total apoptotic cells (early apoptotic + late apoptotic) were calculated using unpaired Mann-Whitney U test in HC vs JSLE comparisons and paired Mann-Whitney U for unstimulated vs IFN-α stimulated comparisons. HC=healthy controls, IFN=interferon, IQR=interquartile range, JSLE=juvenile systemic lupus erythematosus, PBMCs=peripheral blood mononuclear cells, SD=standard deviation.


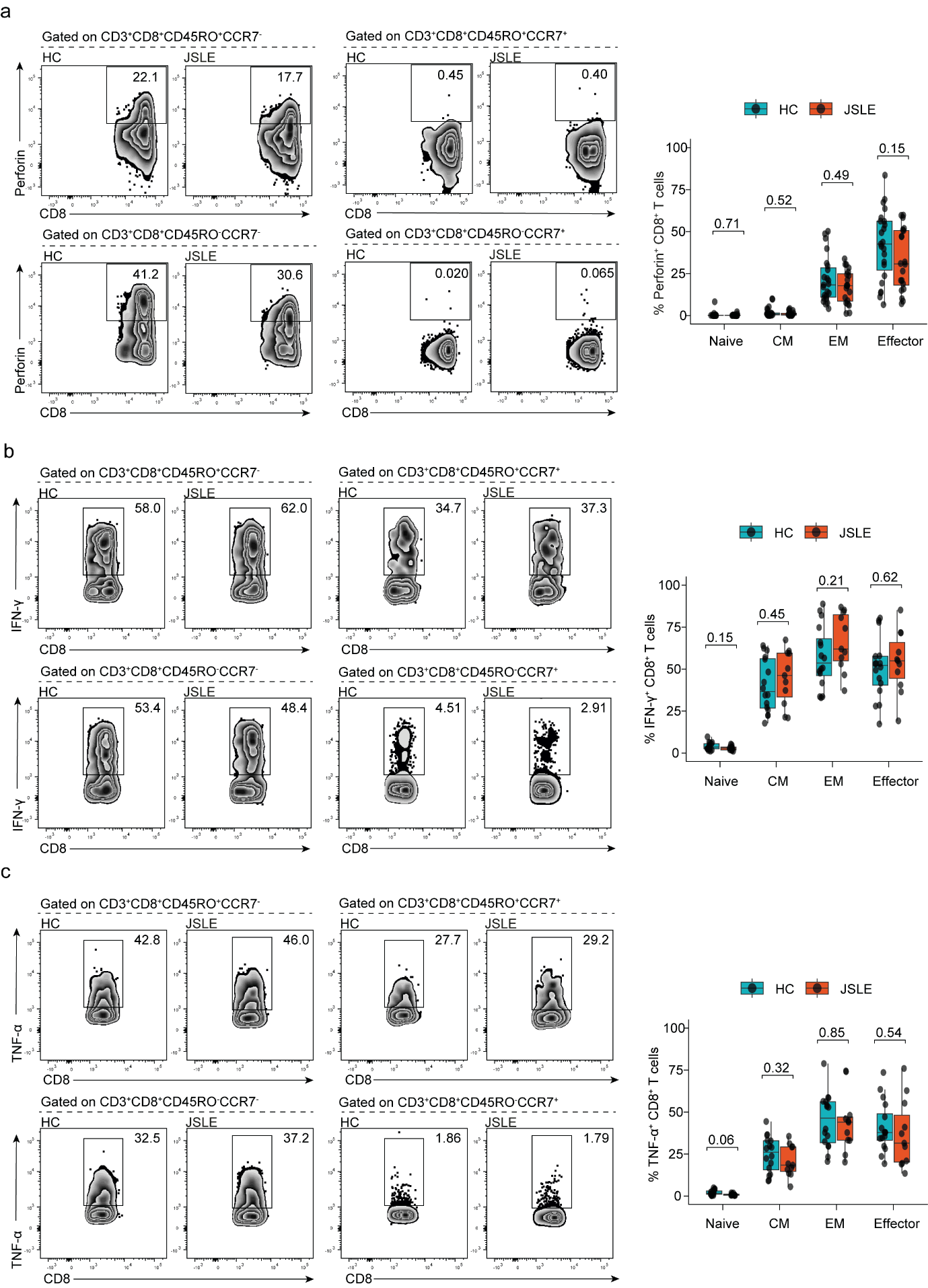


Supplementary Figure S10. No differences in perforin or cytokine expression in CD8^+^ T cell subsets in JSLE vs HC.

Frequencies of **(a)** perforin (HC n=23, JSLE n=20), **(b)** IFN-γ (HC n=16, JSLE n=11), and **(c)** TNF-α (HC n=16, JSLE n=11) in naïve (CD45RO^-^CCR7^+^), central memory (CD45RO^+^CCR7^+^), effector memory (CD45RO^+^CCR7^-^) and effector (CD45RO^-^CCR7^-^), CD8^+^ T cells. Numbers on flow plots indicate percentage of cells. All boxplots show median ± IQR. p-values calculated using unpaired Mann-Whitney U test **(a:** Naïve, CM, EM, **c:** Naïve**)** or t-test **(a:** Effector**, b-c:** CM, EM, Effector, **b:** Naïve**)** as appropriate to distribution of the data. CM=central memory, EM=effector memory, HC=healthy controls, IQR=interquartile range, JSLE=juvenile systemic lupus erythematosus.


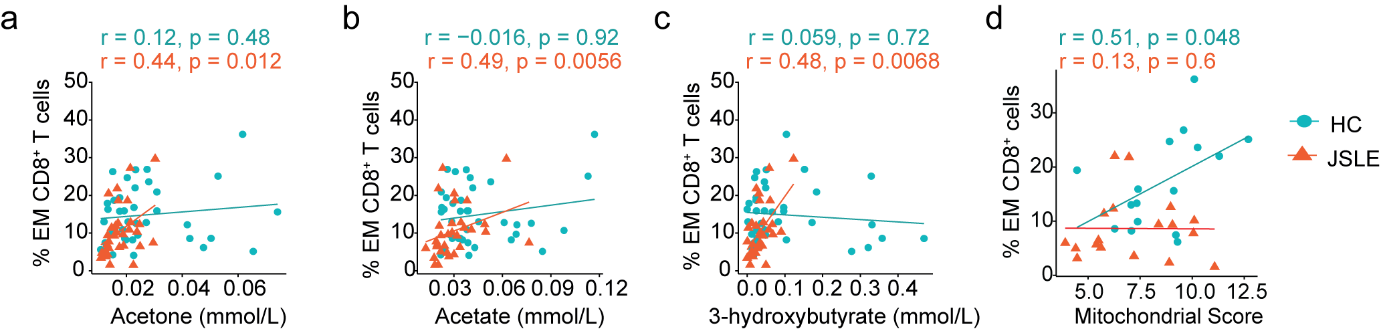


Supplementary Figure S11. Positive correlations between frequencies of EM CD8^+^ T cells and ketone body measurements in JSLE and mitochondrial score in HC.

Scatter plots showing correlations between frequencies of EM CD8^+^ T cells and levels of **(a)** acetone, **(b)** acetate, **(c)** 3-hydroxybuterate in HC (n=38) and JSLE (n=31) and **(d)** mitochondrial score (HC: n=20, JSLE: n=15). Spearman’s rho correlation coefficients and the associated p-values are shown. EM=effector memory, HC=healthy controls, JSLE=juvenile systemic lupus erythematosus.
